# Large-scale spatial drivers of avian schistosomes in Northern Michigan inland lakes

**DOI:** 10.1017/S0031182024000337

**Published:** 2024-04

**Authors:** Jason P. Sckrabulis, Madelyn L. Messner, Jenna Stanny, Ryan B. McWhinnie, Hamzah D. Ansari, Aleena M. Hajek, Alexander Bageris, Thomas R. Raffel

**Affiliations:** 1 Department of Biological Sciences, Oakland University, 118 Library Drive, 374 Dodge Hall, Rochester, MI 48309, USA; 2 Department of Biological Sciences, University of Notre Dame, 100 Galvin Life Science Center, Notre Dame, IN 46556, USA; 3 Department of Chemistry, Oakland University, 146 Library Drive, 260 Mathematics and Science Center, Rochester, MI 48309, USA

**Keywords:** *Lymnaea*, parasites, spatial distribution, swimmer's itch, trematodes, *Trichobilharzia* spp

## Abstract

Avian schistosomes are snail-borne trematode parasites (*Trichobilharzia* spp.) that can cause a nasty skin rash in humans when their cercariae mistake us for their normal bird hosts. We sought to investigate drivers of the spatial distribution of *Trichobilharzia* cercaria abundance throughout Northern Michigan lakes. For 38 sites on 16 lakes, we assessed several dozen potential environmental predictors that we hypothesized might have direct or indirect effects on overall cercaria abundance, based on known relationships between abiotic and biotic factors in wetland ecosystems. We included variables quantifying local densities of intermediate hosts, temperature, periphyton growth rates, human land use and hydrology. We also measured daily abundance of schistosome cercariae in the water over a 5-week period, supported by community scientists who collected and preserved filtered water samples for qPCR. The strongest predictor of cercaria abundance was *Lymnaea* host snail density. *Lymnaea* density was higher in deeper lakes and at sites with more deciduous tree cover, consistent with their association with cool temperature habitats. Contrary to past studies of human schistosomes, we also found a significant negative relationship between cercaria abundance and submerged aquatic vegetation, possibly due to vegetation blocking cercaria movement from offshore snail beds. If future work shows that these effects are indeed causal, then these results suggest possible new approaches to managing swimmer's itch risk in northern MI lakes, such as modifying tree cover and shallow-water vegetation at local sites.

## Graphical abstract

**Figure:**
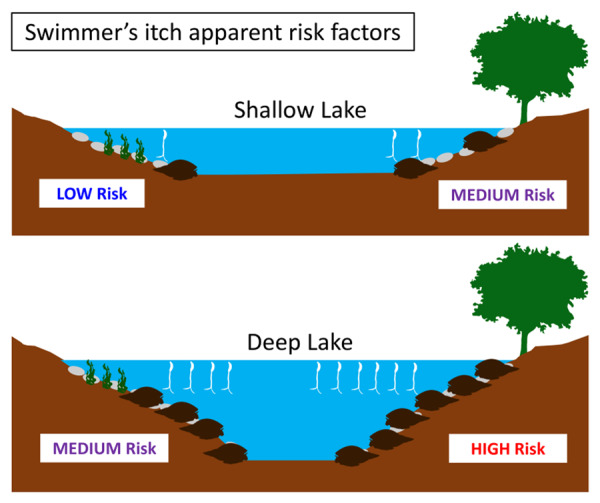

## Introduction

Cercarial dermatitis, also known as ‘swimmer's itch’ (SI), is a rash caused by avian schistosomes, snail-borne parasites related to the causative agents of human schistosomiasis (*Schistosoma* spp.; Brant and Loker, 2009; Colley *et al*., 2014; Horák *et al*., 2015). Avian schistosomes are a diverse group of trematode (flatworm) parasites, mostly in the genus *Trichobilharzia*, that normally use water birds as their definitive hosts (Brant and Loker, 2009). Infected snails release infectious free-living larvae, known as cercariae, which swim through the water in search of a bird to infect. Avian schistosomes cannot complete their life cycles in humans, but their cercariae sometimes mistake humans for birds and penetrate our skin resulting in the distinctive rash (Verbrugge *et al*., 2004*a*; Horák *et al*., 2015). Anecdotal evidence suggests that SI is increasing in Michigan inland lakes, where it discourages swimming and other recreational activities impacting the local economy during the summer (Muzzall *et al*., 2003; Verbrugge *et al*., 2004*b*; McPhail *et al*., 2022; Soper *et al*., 2023). The drivers for distribution of human schistosomes have been widely studied at multiple spatial scales (Yang *et al*., 2006; Brooker, 2007; Zhou *et al*., 2011); however, ecological drivers of among-lake variation in avian schistosomes have been relatively understudied (McMullen and Brackett, 1948; Rudko *et al*., 2018; Soper *et al*., 2023).

**Figure 1.**
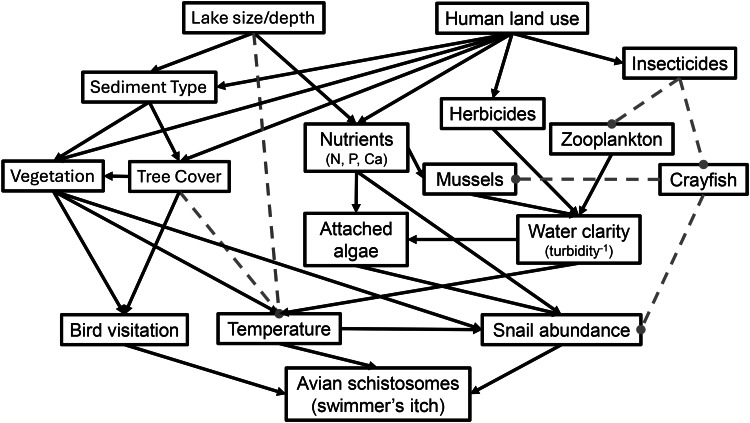
*A priori* hypothesized drivers of snail and avian schistosome abundance in northern Michigan lakes. Solid lines represent positive effects, and dashed lines represent negative effects.

Prior studies on schistosomes and other trematode parasites show that their distributions closely match the distributions of their molluscan intermediate hosts (Skírnisson *et al*., 2004; Dida *et al*., 2014; Marszewska *et al*., 2016; Gordy *et al*., 2018; Soper *et al*., 2023). Therefore, environmental factors that influence the abundance of intermediate host snails are likely to be major predictors of cercaria abundance (see Fig. 1. for *a priori* hypothesized relationships; Rohr *et al*., 2008*a*; Paull *et al*., 2015). Studies on other trematode parasites have shown that pollution from agricultural chemicals including fertilizer, herbicides and insecticides can all increase the densities of snail intermediate hosts, leading to higher infection rates (Rohr *et al*., 2008*a*, 2008*b*; Halstead *et al*., 2014). These chemicals typically act by either increasing growth of attached algae (periphyton) eaten by snails by clearing the water of phytoplankton, increasing water clarity and light penetration (Johnson and Chase, 2004; Brown and Lydeard, 2009) or killing off snail predators (e.g. crayfish; Rohr *et al*., 2008*a*; Halstead *et al*., 2014; Halstead *et al*., 2018). However, water clarity might also be altered by invasive zebra or quagga mussels (*Dreissena* spp.) that filter algae out of the water column (Geisler *et al*., 2016). Other potentially important factors for snail abundance include habitat characteristics such as wave action, water depth, lake size, abundance of submerged vegetation, abundance of snail predators (crayfish) or the availability of solid substrates (Laman *et al*., 1984; Dida *et al*., 2014; Rohr *et al*., 2023). For example, one of the strongest positive predictors of host snails for human schistosomes is the abundance of aquatic vegetation, likely due to the increased surface area available for periphyton growth (Underwood *et al*., 1992; Wood *et al*., 2019; Rohr *et al*., 2023). Based on the results of these past studies, we predicted greater abundance of host snails (namely *Lymnaea*, *Planorbella* and *Physa* spp. snails; Blankespoor and Reimink, 1991; Muzzall *et al*., 2003; McPhail *et al*., 2021), and thus avian schistosome cercariae (namely *Trichobilharzia* spp.; McPhail *et al*., 2021; Rudko *et al*., 2022; Soper *et al*., 2023), in sites with higher water clarity (the inverse of turbidity), faster growth of attached algae, more submerged vegetation, higher nutrient loading in water or sediment and agricultural or urban land use at the local or watershed scales (Fig. 1).

However, cercaria abundance at any given site is not solely driven by host snail abundance. Temperature has long been known to have direct positive effects on rates of parasite development and cercaria production from snails for a variety of trematode species (McCreesh and Booth, 2014; Paull *et al*., 2015; Nguyen *et al*., 2021). Furthermore, snails typically have higher rates of development, reproduction and population growth at warmer temperatures (Nguyen *et al*., 2021). However, it is important to recognize that temperature can have complex and non-linear effects on snail and trematode biology depending on the temporal scale investigated (e.g. thermal stress; Paull *et al*., 2015). Trematode-infected snails also have higher rates of cercaria production when they have access to higher quality food sources (Civitello *et al*., 2018). Cercaria abundance may also be driven by definitive bird host visitation to the lake, which should increase infection prevalence in populations of intermediate host snails (Muzzall *et al*., 2003; Byers *et al*., 2008). All of these factors may lead to more cercaria being produced and released into the water (Fig. 1). Lastly, cercaria abundance at a particular location could be impacted by factors that influence cercaria distributions within a lake, such as water currents and related variables like wind, wave action, effective fetch (the distance over which wind can travel across open water as a measure of potential wave action and surface currents), wind speed and wind direction (Fig. 1). For example, onshore wind and water currents have been found to increase cercaria abundance at local beaches, presumably by bringing in cercariae that were released off-site into the local environment (Upatham, 1974; Muzzall *et al*., 2003; Rudko *et al*., 2018; Sckrabulis *et al*., 2020).

**Figure 2.**
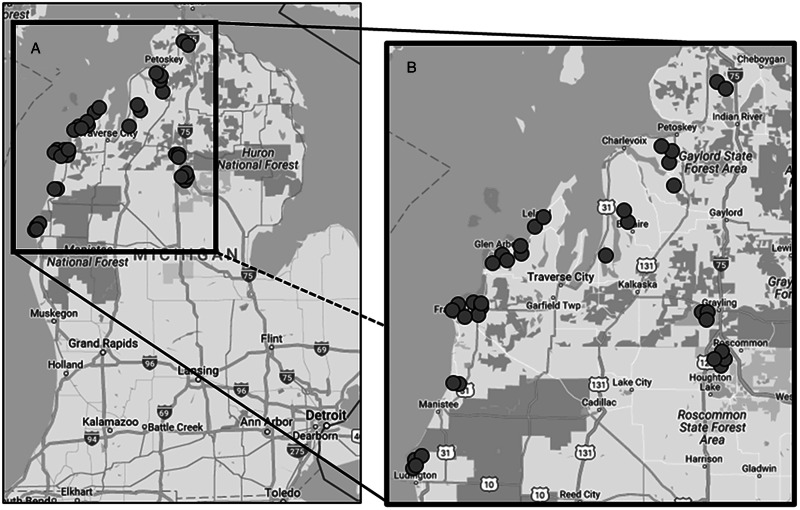
Map of survey sites in northern Michigan. Panel A depicts all sites at the state level, and panel B depicts all sites as an inset.

We conduct an exploratory analysis investigating potential drivers of avian schistosome abundance in northern Michigan inland lakes by collaborating with a large network of local volunteers to survey 16 lakes throughout northern MI in the summer of 2016 (Fig. 2; Table S1). We focused our study on summer months, at the time of peak human recreational water use (i.e. swimming) and reported SI incidence in this region (Sckrabulis *et al*., 2020). We also quantified several dozen environmental predictors hypothesized *a priori* to influence cercaria and host snail abundance, based on known relationships between various abiotic and biotic factors in wetland ecosystems (Fig. 1; Table 1). Here, we take an analytical approach similar to Rohr *et al*. (2008*b*), where we measured a large number of environmental factors that could potentially influence our system but restricted models to only include plausible predictors of individual response variables. We used stepwise model selection with multiple linear regression and generalized linear mixed effects models to conduct an exploratory analysis of hypothesized predictors of cercaria abundance and host snail abundance.

**Table 1.** Variables included in between-site analyses of avian schistosome abundance and the best predictors of that abundance

Hypothesized drivers of avian schistosome concentration	
Potential intermediate hosts (count / m^2^)	Water and sediment chemistry
*Lymnaea* spp. Density	Inorganic nitrogen (mg N / L)
*Physa* spp. Density	Orthophosphate (mg P / L)
*Planorbella* spp. Density	Sediment phosphorus (mg P / g soil)
All combinations of hosts (e.g. *Lymnaea* + *Physa*)	2-4-D concentration (ppb)
Lake level characteristics	Turbidity (NTU)
Surface area (acres)^a^	Alkalinity (ppm CaCO_3_)
Maximum depth (feet)^a^	Riparian site characteristics (index)
Land:water ratio^a^	Woody cover
Temperature (Celsius)	Grassy cover
Zooplankton (count)	Barren cover
Periphyton growth rate (Chl-a RFU / 3 wks)	Tree cover
Bird observations (count)	Road cover
Zebra mussels	Park/beach cover
Biomass on sampler (g)	Dock cover
Number on sampler (count)	Buildings (presence/absence)
Density (count / m^2^)	Littoral site characteristics
Crayfish abundance (count)	Sediment (index of each type)
Land use characteristics (within 1 mile & watershed)	Vegetation (index of each type)
% Urbanized^a^	Slope (meters to 30 and 60 cm depth)
% Cropland^a^	Effective fetch (kilometer)
% Pasture^a^	Wave action (index)
% Forest^a^	

a Predictor variable that was quantified at the lake level instead of separately for each site.

## Materials and methods

### Sampling sites and times

We sampled at 38 sites on 16 inland lakes in northwestern Michigan (Fig. 1, Table S1). Site locations were largely determined by stakeholder interest due to each lake individually funding the study on their lake. Despite this, these sites represented a range of lake sizes, shoreline types (e.g. beach *vs* marsh) and levels of human activity. Each sampling site was restricted to a 15 m stretch of shoreline and extended at least 15 m into the littoral zone (out to approximately 0.8 m depth) and 15 m into the riparian zone. We conducted all aquatic sampling within the limits of these zones. Before daily sampling began, 2 HOBO pendant temperature loggers (Onset Computer Corp., Bourne, MA, USA) and a marker buoy were installed at each site at 60 cm depth. Both loggers recorded hourly temperature readings, but 1 logger at each site also recorded hourly light intensity and was anchored to ensure the light sensor remained horizontal and facing upwards. We also placed periphyton samplers at this depth (see Supplemental Materials ‘Field survey methods – Periphyton growth’). One exception was Deer Lake, where the slope was too shallow that we decided to place equipment at only 45 cm depth to avoid going too far outside the 15 × 15 m sampling area. Daily community science sampling occurred during a 28-day period in July and August 2016, starting on July 5 (detailed below). Daily measurements started 2–3 weeks earlier at selected sites on Crystal Lake and Intermediate Lake based on individual lake support. Site visits and snail surveys started the week prior to this sampling period (the week of June 26) and occurred every 2 weeks at most sites (3 total surveys) and weekly at a few selected sites (at least 1 site at each lake; 5 total surveys).

### Community science sampling – daily cercaria samples, weather and bird observations

Cercaria release into the environment is highly variable at a daily time scale for avian schistosomes (Soldánová *et al*., 2022). Therefore, to capture a clear pattern of cercaria abundance at each site, we employed daily sampling utilizing a community science approach. Daily cercaria samples were collected by trained community science volunteers, who were provided with pre-labelled 15 mL centrifuge tubes, a 1 L pitcher, a custom-made 35 *μ*m Nitex mesh filter (Fig. S1), a squirt bottle filled with tap water, another squirt bottle filled with 70% ethanol, a small funnel, and a stand to hold the filter during filtration (if necessary). Cercariae are likely to have aggregated distributions in the water, so to reduce the chances of missing small clumps of cercariae we sought to collect a distributed sample from each sampling site. We therefore asked volunteers to filter 24 separate 1-L scoops of water for each daily sample at each sampling site. Each morning, when *Trichobilharzia* spp. cercariae are released into the environment (~8:00–10:00 AM; Soldánová *et al*., 2016), volunteers entered the water near the left boundary of the site while holding the 1-liter pitcher and the Nitex mesh filter, haphazardly collecting water from the surface using the pitcher and pouring it through the filter as they moved in a zig-zag pattern throughout the littoral zone of the sampling site. Volunteers were allowed to collect their water samples in a bucket prior to filtration, allowing sediment to settle to the bottom of the bucket prior to decanting the water through the mesh filter. Avian schistosome cercariae typically move toward light and stay near the water surface (Cort and Talbot, 1936; Brachs and Haas, 2008), so they are unlikely to have been lost through this procedure. Once the lake water had been filtered, the squirt bottle containing tap water was used to rinse any residual material into 1 corner of the filter, followed by a final rinse with 70% ethanol to wash the sample into a 15 mL centrifuge tube using a small funnel. The use of 70% ethanol killed and preserved the DNA of any cercariae captured in the sample. Volunteers rinsed and back-rinsed their filters daily with tap water to keep the mesh clean. Samples were stored at room temperature until they were transported back to Oakland University at the end of the survey for further processing. Community science volunteers at each site were also encouraged to collect daily data describing weather conditions, air temperature, onshore wind speed and direction with the provided equipment. They were also asked to tally any sightings of birds within visual range of the study site.

### qPCR quantification of avian schistosomes

After the survey was completed, we used qPCR to quantify the number of gene copies of avian schistosome DNA. Due to budgetary limitations, we developed a low-cost protocol for this process. Our protocol is like that of Rudko *et al*. (2018); however, we aimed to develop this protocol to be economical and scalable to a larger number of volunteer-collected samples. Briefly, we used a recently detailed protocol and custom primers that target a highly conserved region of 18S ribosomal RNA gene sequences specific to schistosome parasites (Jothikumar *et al*., 2015). This assay only differentiates schistosomes *vs* non-schistosomes; we assumed that any amplified DNA largely belonged to the genus most associated with swimmer's itch in this geographic region, *Trichobilharzia*, though other SI-causing schistosomes from the genera *Schistosomatium* and *Gigantobilharzia* have also been observed in Michigan (Muzzall *et al*., 2003; McPhail *et al*., 2022; Rudko *et al*., 2022, Soper *et al*., 2023). We decided to divide each field sample in half prior to extraction, which we hoped would allow us to assess potential sources of error in the assay (i.e. extraction error *vs* qPCR error). For each field sample, we added together the qPCR estimates of cercaria abundance for the 2 extractions. qPCR data is typically lognormally distributed, so we log_10_-transformed the daily cercaria estimates and averaged them to obtain log_10_ cercaria abundance for each site. Log_10_ cercaria abundance was used as our proxy for avian schistosome risk for all our among-site analyses. For a full description of this protocol, see the Supplementary Materials (‘Low-cost protocol for avian schistosome DNA detection’).

### Habitat assessment

At the beginning of the sampling period, a team of Raffel Lab student researchers conducted a habitat assessment in the littoral zone (15 × 15 m in water) and the riparian zone (15 × 15 m on land) using a standardized checklist modelled after the Environmental Protection Agency's 2012 National Lakes Assessment protocol (EPA 841-B-11-003). For all components of the site assessment, a numerical score was used to indicate abundance of landscape or substrate types based on the following numeric index: 0 = Absent, 1 = Sparse (<10% coverage), 2 = Moderate (10 − 40% coverage), 3 = Heavy (40 − 75% coverage) and 4 = Very Heavy (>75% coverage). The riparian ground cover was observed, checking for the abundance of vegetation (woody shrubs, saplings, herbs and grasses) and barren areas (dirt/sand/rock). Riparian canopy and human influence were also classified and recorded. Habitat in the littoral zone was assessed by noting the presence/absence and abundance of various substrates (boulder, cobble, gravel, sand, muck) and types of aquatic vegetation (submergent, emergent, floating and total plant cover).

### Visual quadrat surveys

Each site was visited 3–5 times to measure the abundance and diversity of aquatic snails within the littoral zone, which we divided into 3 depth areas (0–40 cm, 40–80 cm and >80 cm). PVC quadrat sampling frames (0.09 m^2^) were haphazardly tossed within each depth zone until a total of 4 frames were counted in each. A clear-bottom viewing bucket was used to observe and count the number of snails on the substrate within the boundaries of each frame. Densities were recorded for snails, visually identified to the genus level in the field. Densities of any other organisms (e.g. crayfish and mussels) present in a given quadrat were also recorded as they were encountered. Snails were also collected from throughout the littoral area and preserved in 70% ethanol to verify the proportion of snails from each species during 15-minute intensive targeted searches after quadrat sampling was completed during each visit. We computed several indices of snail abundance, including total abundance, and both individual densities of each snail type, and combined densities of *Lymnaea*, *Planorbella* and *Physa* snails as potential predictors of cercaria abundance.

### Environmental variables

Alongside the visual quadrat surveys and timed snail collections conducted at the time of each visit, we also collected samples to assess other biotic and abiotic variables that might impact snail or cercaria presence (Fig. 1). We set out several sampling apparatuses to collect mussel settling rates and periphyton/biofilm formation. We collected water and sediment samples to assess water and sediment chemistry. We conducted multiple instances of crayfish trapping and zooplankton sampling. Lastly, we also assessed lake watershed land-use information using GIS. See the Supplementary Materials (‘Field survey methods’) for a full description of methods used for each of these variables.

### Statistical analyses

**Figure 3.**
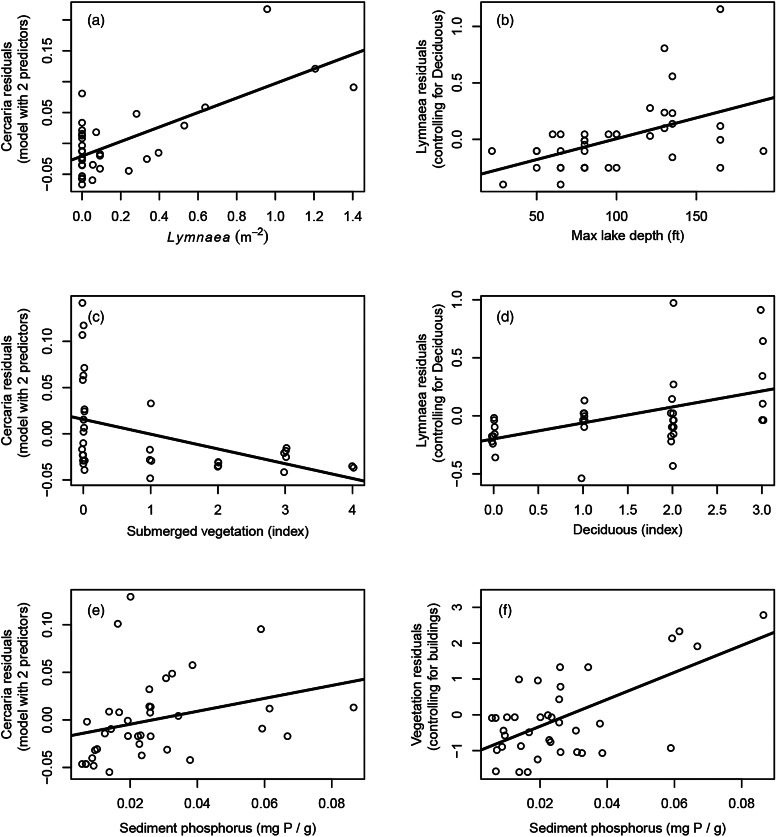
Residual plots for the main predictors of cercaria abundance (Panels A, C, E), Lymnaea density (Panels B & D), and submerged vegetation (Panel F). The top predictors of cercaria abundance were (A) *Lymnaea* spp. density, (C) submerged vegetation, and (E) sediment phosphorus. The top predictors of *Lymnaea* spp. density were (B) maximum lake depth and (D) deciduous tree cover. The top predictors for submerged vegetation were (F) sediment phosphorus and presence of buildings (not shown). All models depict each relationship after accounting for the effects of the other predictors in that model. The raw data relationships can be found in Fig. S2.

To investigate possible relationships between environmental factors (abiotic and biotic) and host snail cercaria abundance, we took an analytical approach similar to that of Rohr *et al*. (2008*b*), where we measured a large number of variables that could plausibly influence our system. There are potential risks of obtaining spurious low *P* values due to model over-fitting (overly complex models) or the presence of influential outliers in analyses that contain many potential predictors relative to sample size (*n* = 38 sites). We sought to reduce these risks by (1) sampling a large number of sites relative to prior studies in this region, (2) limiting the analysis of each response variable to seemingly plausible explanatory variables (Fig. 1), (3) log_10_-transforming count variables and those with skewed distributions, (4) examining each statistical relationship graphically to ensure that it had a plausible directionality and was not driven by influential outliers and (5) limiting the size of individual models to a maximum of 5 predictors during model selection.

All data were aggregated to the site level (*n* = 38), using the average of each measured variable over the entire study period (e.g. average daily log_10_ cercaria abundance over at least 1 month, or average log_10_ snail density across at least 3 quadrat surveys). This resulted in all but a few predictor variables having complete data for all 38 sites (Table S3). We generated a correlation matrix to help identify pairwise relationships between variables, where relationships were considered strong candidates when the correlational statistic r was > 0.4 or < −0.4. A summary of all correlates with response variables of interest is presented in Table S2. We then used the program R (v4.3.1; R Core Team, 2023) to conduct stepwise model selection to identify a set of likely predictor variables for each response variable (linear regression using the ‘regsubsets’ function in the package ‘leaps’; Lumley and Miller, 2020). A list of potential predictors included in the model for each response variable is provided in Table S3. To reduce chances of model over-fitting, we limited the maximum number of variables per ‘regsubsets’ model to 5 (nvmax = 5); because this function only returns models of the size specified, we sorted the list of models returned by the function by adjusted *R*^2^ to assess model fit and predictor inclusion in the model. This generated a set of up to 5 potentially important predictor variables for each response variable. We conducted 2 versions of this analysis for each response variable that included temperature variables as hypothesized predictors, 1 with and 1 without including these predictors, due to missing temperature data from 1 site.

We then combined the top predictor variables identified as potentially important from either ‘regsubsets’ output (between 4 and 9 predictors) into a single model and used manual backward selection to remove non-significant predictors *via* F-tests (P < 0.05). We also considered predictors for possible removal if the effect direction was opposite of the hypothesized effect (see Fig. 2); these were examined on a case by case basis (see Discussion). At this stage, we added 1 additional hypothesized predictor to each relevant model that was not included in the ‘regsubsets’ output due to missing data from 1 site (sediment phosphorus; Table S3), assessed its significance, and again simplified the model *via* backward selection.

Although most predictor variables in this analysis were measured at the level of individual sites, some predictors had only a single value for each lake (e.g. maximum lake depth). We therefore sought to account for potential random effects of Lake in our analysis. The ‘regsubsets’ function only supports linear regression models (i.e. no random effects), and methods allowing for automated stepwise or all-subsets selection of mixed effects models are computationally intensive (e.g. the ‘dredge’ function in the package ‘MuMIn’; Bartón, 2019). We therefore added a random effect of ‘Lake’ to each final model, to guard against variable inclusion due to pseudoreplication (generalized linear mixed effects models using the ‘lmer’ function in the package ‘lme4’; Bates *et al*., 2015). Once we had a final mixed effect model for each response variable (presented in Tables 2 & S4), we tested for spatial confoundment by adding main effects of latitude and longitude to each model, followed by another round of backward selection. If a predictor was kicked out by adding either latitude or longitude to the model, we considered it a spatially confounded predictor.

We verified the robustness of our core model outputs by also utilizing the ‘exhaustive’ method within the ‘regsubsets’ function to conduct an all-subsets analysis for each of the response variables presented in Table 2, again with nvmax = 5. We examined the top 10 models for each response variable in order of adjusted *R*^2^. All-subsets model outputs can be difficult to interpret in analyses containing clusters of highly correlated predictor variables, but this method is less prone to variable ‘trapping’ than stepwise selection procedures. Here we used all-subsets to confirm that each of the primary predictors from our core models, or a closely related predictor, appeared in all or most of the top 10 models for each response variable.

**Table 2.** Final models for each response variable of interest, following stepwise model selection

Response	Predictor	Coef.	F	df	P(F)
Log_10_ cercariae per L	Log_10_ *Lymnaea*	0.12	34.1	1.33	<0.0001
	Submerged vegetation	−0.023	13.3	1.33	0.0009
	Sediment phosphorus^a^	1.0	5.2	1.33	0.0293
Log_10_ *Lymnaea*	Maximum lake depth^b^	0.0037	11.5	1.14	0.0018
	Deciduous index	0.14	9.9	1.21	0.0034
Submerged vegetation	Sediment phosphorus	30.1	17.7	1.19	0.001
	Buildings (present/absent)^c^	0.8	15.0	1.19	0.021

All final models included ‘Lake’ as a random effect. Note that the ‘Anova’ function from the ‘car’ package uses the Kenwood-Roger approximation for estimating degrees of freedom for F-tests, which can result in non-integer values (Fox and Weisberg, 2019).

a Variable with missing data (one missing datapoint for sediment phosphorus)

b Lake-level variable

c Buildings became non-significant when Longitude was added to the final model (spatial autocorrelation)

In general, we are more confident of results where (1) the final model was relatively simple (i.e. 3 or fewer significant predictors), (2) predictors present in the top model were also among the top single correlates of a given response variable, (3) predictors selected by stepwise selection also appeared in the top 10 models in all-subsets analysis, (4) there was no evidence that a particular relationship was driven by spatial confoundment, and (5) there was an obvious mechanistic explanation for strength and direction of an observed statistical relationship.

Note that we also conducted an analysis of potential predictors of zebra mussel abundance (see Sckrabulis, 2020), but this is outside the scope of the current manuscript.

## Results

### General patterns of snail and parasite distribution

Out of the 38 sites sampled in this study, 31 generated positive qPCR results for the presence of avian schistosome cercariae. There was a mean density of 0.05 log_10_ cercariae per liter across all sites. At 30 sites, we detected known host snails of avian schistosomes (15 with *Lymnaea*, 18 with *Physa*, 14 with *Planorbella*). At 4 sites, we detected avian schistosome DNA but did not detect known host snails of any genus.

### Cercaria abundance

The strongest significant predictor of cercaria abundance was the density of *Lymnaea* spp. snails at each site (Log_10_ *Lymnaea*; Table 2; Figure 3A & B). Accounting for the presence of *Physa* or *Planorbella* spp. snails in any way did not increase predictive power of the model. We also found a significant negative effect of submerged aquatic vegetation on cercaria abundance (Table 2; Figure 3C & D). This effect was not driven by influential outliers and was evident with or without including *Lymnaea* spp. abundance in the model (Fig. 3C & D). Furthermore, we were unable to find any alternative variables we measured that correlated with vegetation and might help account for this pattern. After accounting for *Lymnaea* spp. abundance and submerged vegetation, we also detected a significant positive effect of sediment phosphorus (Table 2; Figure 3E & F). This pattern was completely dependent on the effects of the other 2 variables on the model, however, as there was no evidence of a direct correlation between cercaria abundance and sediment phosphorus prior to stepwise model selection (Table S2; Fig. S2). In all-subsets analysis, the top 10 models all contained Log_10_ *Lymnaea* density and sediment phosphorus (Table S6). All 10 top models also contained either submerged vegetation or the highly correlated (and biologically similar) ‘total vegetation’ (Table S6). Neither latitude nor longitude contributed significantly to the final model.

### Snail abundance

The strongest predictors of total snail abundance were turbidity and effective fetch, with snails being more abundant at sites with lower turbidity (high water clarity) and high effective fetch (Table S4). However, the effect of turbidity became non-significant when longitude was added to the model (Table S5). We also found a significant positive effect of local conifer tree abundance on total snail density (Table S4). Total snails at all sites were dominated by *Pleurocera* spp. snails; when we analysed *Pleurocera* and *Lymnaea* spp. independently, it was clear that these 2 genera responded to very different variables, and the statistical effects of turbidity and effective fetch were driven by *Pleurocera* spp. (Table S4). We also detected a positive effect of mean day temperature on *Pleurocera* spp. abundance, but this was only evident in the full model (Table S4). In contrast, the best predictors of *Lymnaea* spp. snail abundance were maximum lake depth and the local abundance of deciduous trees (Table 2; Figure 3B & D). Deciduous tree cover appeared in all 10 top models in the all-subsets analysis (Table S7). Maximum lake depth appeared in 8 of the top 10 models; the remaining 2 models contained ‘lake surface area’ which was highly correlated with maximum lake depth (*r* = 0.88; Table S7). The *Lymnaea* and *Pleurocera* models were not affected by adding latitude or longitude, which were nonsignificant in both models.

### Submerged vegetation

We examined the possible environmental predictors of submerged aquatic vegetation, which was the second-best predictor of cercaria abundance. The best single predictor of submerged vegetation was sediment phosphorus at the site (Table 2; Figure 3F). After accounting for sediment phosphorus, we also found significantly higher vegetation levels at sites with a building present in the riparian zone (Table 2). Per cent forest and per cent cropland in the watershed were also significant predictors at the end of stepwise model selection, but they became non-significant after adding ‘Lake’ as a random effect (Table 2). Sediment phosphorus appeared in all 10 top models in all subsets regression and ‘buildings’ appeared in the top 9 top models (Table S8); however, ‘buildings’ became nonsignificant when longitude was added to the model (Tables 2 & S5).

### Temperature

Temperature correlated strongly with maximum lake depth, which was a strong predictor of *Lymnaea* spp. abundance (Table S2). We therefore examined possible environmental predictors of 3 measures of water temperature: mean (overall average) temperature, mean day temperature (average of daily maxima), and mean night temperature (average of daily minima). We limited these analyses to only include predictors related to lake hydrology (e.g. maximum lake depth) or potential sources of shade (e.g. abundance of trees or aquatic vegetation; Table S3). For all 3 measures, temperature was lower in larger lakes. The best predictors of all 3 temperature indices were either maximum lake depth or lake surface area (Table S4), which were highly correlated with each other. We also detected a significant negative effect of submerged vegetation on mean daytime temperature following stepwise model selection, which might indicate a shading effect; however, this effect became non-significant when a random effect of ‘Lake’ was added to the model (Table S4). None of the temperature models were affected by addition of latitude or longitude, neither of which was a significant predictor in any of these models.

## Discussion

### Cercaria abundance

Consistent with previous studies in northern Michigan, avian schistosome abundance throughout our survey was driven by the density of *Lymnaea* spp. snails, suggesting that these are the most important snail hosts driving variation in *Trichobilharzia* spp. cercaria abundance in these northern Michigan lakes (Blankespoor and Reimink, 1991; Muzzall *et al*., 2003; Rudko *et al*., 2018). *Physa* and *Planorbella* spp. snails are also known hosts of *Trichobilharzia* spp. parasites, but their inclusion in our analysis led to no increase in predictive power. *Physa* and other genera of snails might also harbour *Trichobilharzia* spp. parasites that are implicated in SI in other parts of Michigan (McMullen and Brackett, 1948; McPhail *et al*., 2021), though evidence suggests that *Trichobilharizia* spp. from *Planorbella* snails may contribute to SI risk throughout the state of Michigan (Soper *et al*., 2023). Additionally, the observed abundance of *Physa* and *Planorbella* snails at our local sampling sites might not have always reflected their off-site abundances in the lakes we sampled. Many of our sampling sites were (by necessity) publicly accessible sandy or rocky beach habitats, which tended to be dominated by *Lymnaea* and (non-host) *Pleurocera* snails. However, many were adjacent to more-vegetated sites where *Physa* and *Planorbella* might have been more abundant. Interestingly, we detected relatively high levels of cercariae at some sites where we observed few or no *Lymnaea* spp. snails (Fig. S2). The most likely source of cercariae at these sites is from snails outside of the study site, either in deeper water than we surveyed or along the shore in either direction (Laman *et al*., 1984; Muzzall *et al*., 2003). Past studies have suggested that cercaria are transported from off-site *via* waves or water currents (Upatham, 1974; Leighton *et al*., 2000; Muzzall *et al*., 2003; Rudko *et al*., 2018; Sckrabulis *et al*., 2020). We found no effect of bird abundance, at least not based on sightings by our community scientists during the study period. This result might suggest that local abundance of definitive host birds might not be a limiting factor for sustaining parasite populations in these lakes, consistent with a past study (Rudko *et al*., 2022). However, bird visitation likely varies seasonally, and bird visitation data from earlier in the season might have been more predictive of late-summer cercaria production by infected snails due to the time it takes for snails to develop patent infections.

We also found a negative effect of submergent vegetation on cercaria abundance in our final model (Table 2; Figure 3C). This result is opposite a pattern widely reported for human schistosomes, where aquatic vegetation is sometimes the strongest environmental predictor of human schistosomiasis risk due to providing habitat for *Biomphalaria* and *Bulinus* host snails (Klumpp and Chu, 1980; Boelee and Laamrani, 2004; Rohr *et al*., 2023). The observed negative relationship in the current study was consistent across the range of vegetation levels we quantified, and it was robust to whether *Lymnaea* spp. abundance was included as a covariate (*F*_1,36_ = 5.81, *P*(F) = 0.021). The consistency of this result suggests that it was not simply a spurious correlation. Furthermore, the *Lymnaea* host snails most associated with cercaria abundance in the current study have very different habitat preferences than host snails for human schistosomes, preferring silty, sandy, or rocky substrates and cool deep-water habitats (Laman *et al*., 1984). Thus, it is plausible that vegetation has a different effect in this system. We explored possible *a posteriori* hypotheses to explain a negative relationship between avian schistosome cercariae and submerged vegetation in this system. We found literature support for 3 alternative mechanisms (Warren and Peters, 1968; Gibson and Warren, 1970; Christensen, 1979). First, Warren and Peters (1968) found that some species of plants can act as accidental hosts for schistosome cercariae, which might remove cercariae from the water column. However, only a small proportion of the species tested were penetrated by cercariae, and none of those species are native to Michigan lakes (Warren and Peters, 1968). Second, Gibson and Warren (1970) showed that bladderworts (genus *Utricularia*), a native carnivorous plant common in MI lakes, is an effective predator of schistosome cercariae and can rapidly reduce cercaria abundance in water. However, we did not collect data on the species identities of aquatic plants in our study, making it difficult to evaluate the plausibility of this hypothesis. Third, Christensen (1979) found that multiple types of floating vegetation can effectively block cercaria movement (Christensen, 1979). Such an effect might plausibly prevent cercariae movement into a beach area from off site, especially for avian schistosome cercariae that tend to swim toward the water surface in search of floating (duck) hosts (Feiler and Haas, 1988). This might also explain why the aquatic vegetation pattern was especially strong at sites with few *Lymnaea* spp. host snails, meaning most of the cercariae detected at these sites probably came from outside the study area. This idea is also consistent with studies showing that onshore wind is an important predictor of daily variation in swimmer's itch risk, presumably due to off-site cercariae being transported towards shore in water currents generated by onshore wind (Rudko *et al*., 2018; Sckrabulis *et al*., 2020).

We found that the best predictor of submerged vegetation was levels of phosphorus in the sediment (Fig. 3F), which is plausible as phosphorus is believed to be a limiting nutrient for aquatic plant growth in northern MI lakes (Bole and Allan, 1978). The presence of an artificial structure (‘buildings’) was a positive predictor of vegetation in the final model (Table 2), but this pattern was spatially confounded with a significant longitude effect. We also discounted an apparent positive effect of 2,4-D herbicide during stepwise model selection, since it seemed unlikely that higher herbicide concentrations led to greater vegetation growth. The reverse causal direction seemed more plausible, i.e. that locals apply 2,4-D in response to increased vegetation.

To our knowledge, ours is the first field study to detect a negative effect of aquatic vegetation on SI-causing cercaria abundance. However, relatively few field studies have quantified cercaria abundance directly from water samples, so we cannot rule out the possibility that this effect might be common. We did not distinguish specific types of vegetation, so we cannot draw conclusions about the effects of specific aquatic plant species. Nevertheless, our results suggest that managing aquatic plant life near beaches might provide promising new ways to help control swimmer's itch in MI lakes. At minimum, we advise caution for lake managers involved in controlling aquatic weeds to discern target weeds from potentially beneficial native plants like bladderwort, which can be easily confused with invasive plants like Eurasian milfoil. Lake managers might also consider retaining existing beds of floating aquatic vegetation located adjacent to swim sites in temperate inland lakes, especially in locations where SI risk is known to be associated with onsite wind (Sckrabulis *et al*., 2020), since floating vegetation may help to reduce the influx of cercariae produced off site. This would be similar to other proposed management strategies aimed at blocking the influx of SI-causing cercariae, such as the floating barriers suggested by Muzzall *et al*. (2003) and R. L. Reimink (personal communication). However, given the positive association between aquatic vegetation and human schistosomiasis risk (Klumpp and Chu, 1980; Boelee and Laamrani, 2004; Rohr *et al*., 2023), more work is needed to verify the extent to which submerged vegetation is associated with reduced SI risk in temperate lakes outside of the current study area.

We also found a significant direct positive effect of sediment phosphorus on cercaria abundance, though this was only a significant predictor after accounting for effects of host snail abundance and submerged vegetation (Fig. 3E & F). This pattern is consistent with prior studies that have found positive bottom-up effects of phosphorus on snail populations, mediated by increased availability of periphyton (Johnson *et al*., 2007; Rohr *et al*., 2008*b*). Since the observed effect of phosphorus was only evident after controlling for local *Lymnaea* abundance, it may be driven by increased rates of cercaria production by individual snails with better nutrition (Civitello *et al*., 2018).

### Snail abundance

We found significant effects of turbidity and effective fetch on the total snail abundance at each site, as well as an unexpected negative effect of local conifer abundance (Table 2). The observed positive relationship between water clarity (the inverse of turbidity) and snail abundance is consistent with our *a priori* hypothesis that increased water clarity would increase growth rates of the periphyton eaten by snails, thereby supporting greater snail population sizes. However, this turbidity effect on total snail abundance was spatially confounded and became nonsignificant when longitude was added to the model. Furthermore, most of the snail communities sampled in this survey were dominated by *Pleurocera* spp. snails, which are not known to harbour SI-causing avian schistosomes (Blankespoor and Reimink, 1991). The best predictors of *Pleurocera* spp. abundance were water clarity and fetch, plus a positive effect of mean day temperature (Table S4), and the numerical dominance of this snail genus was the primary reason for significant effects of turbidity and fetch on total snail abundance in our survey. These patterns are consistent with *Pleurocera* spp. life history, which live at lower (i.e. warmer) latitudes, have thick shells to resist damage from wave action, and are commonly found in the shallow-water zone of larger rivers and lakes (Clarke, 1981; Tiemann *et al*., 2011; Dillon *et al*., 2013).

*Lymnaea* spp. snail abundance was primarily associated with deeper lakes and sites with higher deciduous tree abundance (Fig. 3B & D). All *Lymnaea* spp. snails observed or collected in this survey appeared to fall within the *Lymnaea catescopium* species complex ( = *Stagnicola emarginata*; Correa *et al*., 2010), which is thought to prefer hard substrate (rock/cobble) habitats in colder, deeper-water lakes (Clarke, 1981; Laman *et al*., 1984). Compared to other species, *L. catescopium* is found in deeper water, often at depths greater than 8 m (Clarke, 1981). Our quadrat surveys only extended to approx. 80 cm depth, so *Lymnaea* spp. abundance at our sites is likely underestimated, especially in locations where these snails might be restricted to deeper water. Additionally, *L. catescopium* is typically found at higher latitudes (>40°N) with Michigan being at the southern end of their range (Clarke, 1981), so it is thought to be more adapted to colder temperatures. In our survey, all 3 temperature measures (daytime, nighttime and daily mean temperature) were lower in lakes with greater surface area or maximum depth. It seems plausible that the observed positive relationship between *Lymnaea* abundance and lake depth was driven in part by the availability of deep-water habitats with cooler water temperatures, despite not detecting significant direct effects of local (site-level) water temperature measurements in our analysis.

To our knowledge, this is the first study to detect a relationship between deciduous shade trees and *Lymnaea* spp. abundance, and it would be necessary to conduct further study to determine whether this relationship might be causal. Nevertheless, this positive association might also be plausibly driven by providing definitive bird host habitat or a preference for cool temperatures by *Lymnaea* spp. snails (Fig. S2C & D). During the day when we conducted our surveys, *Lymnaea* spp. snails might only remain in the shallows if there are enough shade trees available to keep the water cool. We did not detect a clear effect of deciduous trees on local water temperature in our analysis, but our temperature measurements were taken at approximately 60 cm depth. Any effects of tree shade on water temperature are more likely to occur in even shallower near-shore water, where we sampled snails but did not collect temperature data.

## Conclusions

It is important to emphasize that all patterns reported in this study are correlational, and follow-up experimental work would be needed to demonstrate causality. This is particularly true for novel findings, such as the apparently negative effect of vegetation on cercaria abundance, or the apparently positive effect of deciduous tree cover on local *Lymnaea* spp. abundance. It is also likely that the importance of some variables might depend on temporal or spatial scale. It remains possible that long-term changes in water clarity might have influenced *Lymnaea* populations in MI lakes over the last few decades, but if this effect was uniform across the study area, then our spatial survey would not have detected it.

Another common problem in ecological systems is that effects can be highly context dependent. For example, the most important drivers of snail abundance in northern MI lakes might differ from the most important drivers in southern MI lakes where the dominant parasite species appears to be a recently described avian schistosome that uses *Planorbella* snails as an intermediate host (McPhail *et al*., 2021; Soper *et al*., 2023). Prior studies found evidence of resource limitation in some northern *Lymnaea* populations (Cuker, 1983; O'Brien *et al*., 2005), suggesting that growth of attached algae (and by extension water clarity and/or nutrients) might sometimes be important drivers of these snail populations. Other studies have found an important role for top-down regulation of *Lymnaea* populations by fish and invertebrate predators (Hershey, 1990; Merrick *et al*., 1991), which we were unable to assess in this study. Future studies are needed to expand the geographical scope of our knowledge about SI drivers in temperate inland lakes and use manipulative experiments to test whether the relationships reported here are likely to be causal.

While this study didn't directly test methods for reducing avian schistosome abundance, identifying environmental predictors of risk does provide information that people could use to inform or develop risk management strategies. In particular, our results indicate that local vegetation (i.e. tree cover and submerged vegetation) might cercaria abundance at the site level. These are factors that could be easily modified by local landowners. If future studies confirm that these relationships are causal, this could open up new possibilities for managing swimmer's itch in inland lakes.

## Supporting information

Sckrabulis et al. supplementary material Sckrabulis et al. supplementary material

## Data Availability

All code and data will be made available on GitHub upon acceptance: https://www.github.com/jasonsckrabulis/sckrabulis_etal_si_survey_2016.

## Supplementary material

The supplementary material for this article can be found at https://doi.org/10.1017/S0031182024000337.

## References

[ref1] Bartón K (2019) MuMIn: Multi-Model Inference [Computer software]. https://CRAN.R-project.org/package=MuMIn

[ref2] Bates D, Maechler M, Bolker B and Walker S (2015) Fitting linear mixed-effects models using lme4. Journal of Statistical Software 67, 1–48.

[ref3] Blankespoor HD and Reimink RL (1991) The control of swimmer's itch in Michigan: past, present, and future. Michigan Academician 24, 7–23.

[ref4] Boelee E and Laamrani H (2004) Environmental control of schistosomiasis through community participation in a Moroccan oasis. Tropical Medicine & International Health 9, 997–1004. 15361113 10.1111/j.1365-3156.2004.01301.x

[ref5] Bole JB and Allan JR (1978) Uptake of phosphorus from sediment by aquatic plants, Myriophyllum spicatum and Hydrilla verticillata. Water Research 12, 353–358.

[ref6] Brachs S and Haas W (2008) Swimming behavior of Schistosoma mansoni cercariae: responses to irradiance changes and skin attractants. Parasitology Research 102, 685–690. 18157546 10.1007/s00436-007-0812-4

[ref7] Brant SV and Loker ES (2009) Molecular systematics of the avian schistosome genus *Trichobilharzia* (Trematoda: Schistosomatidae) in North America. Journal of Parasitology 95, 941–963. 20049999 10.1645/GE-1870.1 PMC2922959

[ref8] Brooker S (2007) Spatial epidemiology of human schistosomiasis in Africa: risk models, transmission dynamics and control. Transactions of the Royal Society of Tropical Medicine and Hygiene 101, 1–8. 17055547 10.1016/j.trstmh.2006.08.004 PMC1975763

[ref9] Brown, KM and Lydeard S (2009) Mollusca: gastropoda. In Thorp JH and Covich AP (eds), Ecology and Classification of North American Freshwater Invertebrates, 3rd Edn. Cambridge, MA, USA: Academic Press, pp. 277–306.

[ref10] Byers JE, Blakeslee AMH, Linder E, Cooper AB and Maguire TJ (2008) Controls of spatial variation in the prevalence of trematode parasites infecting a marine snail. Ecology 89, 439–451. 18409433 10.1890/06-1036.1

[ref11] Christensen NO (1979) *Schistosoma mansoni*: interference with cercarial host-finding by various aquatic organisms. Journal of Helminthology 53, 7–14. 458134 10.1017/s0022149x00005678

[ref12] Civitello DJ, Fatima H, Johnson LR, Nisbet RM and Rohr JR (2018) Bioenergetic theory predicts infection dynamics of human schistosomes in intermediate host snails across ecological gradients. Ecology Letters 21, 692–701. 29527787 10.1111/ele.12937

[ref13] Clarke AH (1981) The Freshwater Molluscs of Canada. Ottawa, Canada: National Museum of Natural Sciences, National Museums of Canada.

[ref14] Colley DG, Bustinduy AL, Secor E and King CH (2014) Human schistosomiasis. Lancet (London, England) 383, 2253–2264. 24698483 10.1016/S0140-6736(13)61949-2 PMC4672382

[ref15] Correa AC, Escobar JS, Durand P, Renaud F, David P, Jarne P, Pointier JP and Hurtrez-Bousses S (2010) Bridging gaps in the molecular phylogeny of the Lymnaeidae (Gastropoda: Pulmonata), vectors of Fascioliasis. BMC Evolutionary Biology 10, 1–12. 21143890 10.1186/1471-2148-10-381 PMC3013105

[ref16] Cort WW and Talbot SB (1936) Studies on schistosome dermatitis. III. Observations on the behavior of the dermatitis-producing schistosome cercariae. American Journal of Hygiene 23, 385–396.

[ref17] Cuker BE (1983) Competition and coexistence among the grazing snail *Lymnaea*, Chironomidae, and microcrustacea in an Arctic epilithic lacustrine community. Ecology 64, 10–15.

[ref18] Dida GO, Gelder FB, Anyona DN, Matano A, Abuom PO, Adoka SO, Ouma C, Kanangire CK, Owuor PO and Ofulla AVO (2014) Distribution and abundance of schistosomiasis and fascioliasis host snails along the Mara river in Kenya and Tanzania. Infection Ecology and Epidemiology 4, 24281. 10.3402/iee.v4.24281 PMC4216393 25405008

[ref19] Dillon RT, Jacquemin SJ and Pyron M (2013) Cryptic phenotypic plasticity in populations of the freshwater prosobranch snail *Pleurocera canaliculata*. Hydrobiologia 709, 117–127.

[ref20] Feiler W and Haas W (1988) Host-finding in *Trichobilharzia ocellata* cercariae: swimming and attachment to the host. Parasitology 96, 493–505. 3405636 10.1017/s0031182000080136

[ref21] Fox J and Weisberg S (2019) An R Companion to Applied Regression, 3rd Edn. Thousand Oaks, CA, USA: Sage.

[ref22] Geisler ME, Rennie MD, Gillis DM and Higgins SN (2016) A predictive model for water clarity following dreissenid invasion. Biological Invasions 18, 1989–2006.

[ref23] Gibson M and Warren KS (1970) Capture of *Schistosoma mansoni* miracidia and cercariae by carnivorous aquatic vascular plants of the genus *Utricularia*. Bulletin of the World Health Organization 42, 833–835. 5311069 PMC2427479

[ref24] Gordy MA, Cobb TP and Hanington PC (2018) Swimmer's itch in Canada: a look at the past and a survey of the present to plan for the future. Environmental Health 17, 73. 30359259 10.1186/s12940-018-0417-7 PMC6203143

[ref25] Halstead NT, McMahon TA, Johnson SA, Raffel TR, Romansic JM, Crumrine PW and Rohr JR (2014) Community ecology theory predicts the effect of agrochemical mixtures on aquatic biodiversity and ecosystem properties. Ecology Letters 17, 932–941. 24811760 10.1111/ele.12295

[ref26] Halstead NT, Hoover CM, Arakala A, Civitello DJ, De Leo GA, Gambhir M, Johnson SA, Jouanard N, Loerns KA, McMahon TA, Ndione RA, Nguyen K, Raffel TR, Remais JV, Riveau G, Sokolow SH and Rohr JR (2018) Agrochemicals increase risk of human schistosomiasis by supporting higher densities of intermediate hosts. Nature Communications 9, 837. 10.1038/s41467-018-03189-w PMC5826950 29483531

[ref27] Hershey AE (1990) Snail populations in Arctic lakes: competition mediated by predation? Oecologica 82, 26–32. 10.1007/BF00318529 28313133

[ref28] Horák P, Mikeš L, Lichtenbergová L, Skála V, Soldánová M and Brant SV (2015) Avian schistosomes and outbreaks of cercarial dermatitis. Clinical Microbiology Reviews 28, 165–190. 25567226 10.1128/CMR.00043-14 PMC4284296

[ref29] Johnson PTJ and Chase JM (2004) Parasites in the food web: linking amphibian malformations and aquatic eutrophication. Ecology Letters 7, 521–526.

[ref30] Johnson PTJ, Chase JM, Dosch KL, Hartson RB, Gross JA, Larson DJ, Sutherland DR and Carpenter SR (2007) Aquatic eutrophication promotes pathogenic infection in amphibians. Proceedings of the National Academy of Sciences 104, 15781–15786. 10.1073/pnas.0707763104 PMC2000446 17893332

[ref31] Jothikumar N, Mull BJ, Brant SV, Loker ES, Collinson J, Secor WE and Hill VR (2015) Real-time PCR and sequencing assays for rapid detection and identification of avian schistosomes in environmental samples. Applied and Environmental Microbiology 81, 4207–4215. 25862226 10.1128/AEM.00750-15 PMC4524150

[ref32] Klumpp RK and Chu KY (1980) Importance of the aquatic weed *Ceratophyllum* to transmission of *Schistosoma haematobium* in the Volta Lake, Ghana. Bulletin of the World Health Organization 58, 791–798. 6975187 PMC2395977

[ref33] Laman TG, Boss NC and Blankespoor HD (1984) Depth distribution of seven species of gastropods in Douglas Lake, Michigan. The Nautilus 98, 20–25.

[ref34] Leighton BJ, Zervos S and Webster JM (2000) Ecological factors in schistosome transmission, and an environmentally benign method for controlling snails in a recreational lake with a record of schistosome dermatitis. Parasitology International 49, 9–17. 10729712 10.1016/s1383-5769(99)00034-3

[ref35] Lumley T and Miller A (2020) leaps: Regression subset selection [Computer software]. https://CRAN.R-project.org/package=leaps

[ref36] Marszewska A, Cichy A, Heese T and Żbikowska E (2016) The real threat of swimmer's itch in anthropogenic recreational water body of the Polish lowland. Parasitology Research 115, 3049–3056. 27083184 10.1007/s00436-016-5060-z PMC4958134

[ref37] McCreesh N and Booth M (2014) The effect of increasing water temperatures on *Schistosoma mansoni* transmission and *Biomphalaria pfeifferi* population dynamics: an agent-based modelling study. PLoS ONE 9, e101462. 24987963 10.1371/journal.pone.0101462 PMC4079709

[ref38] McMullen DB and Brackett S (1948) Studies on schistosome dermatitis X. Distribution and epidemiology in Michigan. American Journal of Hygiene 47, 259–270. 18861396 10.1093/oxfordjournals.aje.a119204

[ref39] McPhail BA, Rudko SP, Turnbull A, Gordy MA, Reimink RL, Clyde D, Froelich K, Brant SV and Hanington PC (2021) Evidence of a putative novel species of avian schistosome infecting *Planorbella trivolvis*. The Journal of Parasitology 107, 89–97. 33556182 10.1645/20-74

[ref40] McPhail BA, Froelich K, Reimink RL and Hanington PC (2022) Simplifying schistosome surveillance: using molecular cercariometry to detect and quantify cercariae in water. Pathogens (Basel, Switzerland) 11, 565. 35631086 10.3390/pathogens11050565 PMC9146278

[ref41] Merrick GW, Hershey AE and McDonald ME (1991) Lake trout (Salvelinus-Namaycush) control of snail density and size distribution in an Arctic lake. Canadian Journal of Fisheries and Aquatic Sciences 48, 498–502.

[ref42] Muzzall PM, Burton TM, Snider RJ and Coady NR (2003) Occurrence, Distribution and Control of the Parasites That Cause Swimmer's Itch in Michigan. Extension Bulletin WQ 58. East Lansing, MI, USA: Michigan State University Extension.

[ref43] Nguyen KH, Boersch-Supan PH, Hartman RB, Mendiola SY, Harwood VJ, Civitello DJ and Rohr JR (2021) Interventions can shift the thermal optimum for parasitic disease transmission. Proceedings of the National Academy of Sciences 118, e2017537118. 10.1073/pnas.2017537118 PMC7980429 33836584

[ref44] O'Brien WJ, Barfield M, Bettez N, Hershey AE, Hobbie JE, Kipphut G, Kling G and Miller MC (2005) Long-term response and recovery to nutrient addition of a partitioned Arctic lake. Freshwater Biology 50, 731–741.

[ref45] Paull SH, Raffel TR, LaFonte BE and Johnson PTJ (2015) How temperature shifts affect parasite production: testing the roles of thermal stress and acclimation. Functional Ecology 29, 941–950.

[ref46] R Core Team (2023) R: A Language and Environment for Statistical Computing [Computer Software]. Vienna, Austria: R Foundation for Statistical Computing.

[ref47] Rohr JR, Raffel TR, Sessions SK and Hudson PJ (2008a) Understanding the net effects of pesticides on amphibian trematode infections. Ecological Applications 18, 1743–1753. 18839768 10.1890/07-1429.1

[ref48] Rohr JR, Schotthoefer AM, Raffel TR, Carrick HJ, Halstead NT, Hoverman JT, Johnson CM, Johnson LB, Lieske C, Piwoni MD, Schoff PK and Beasley VR (2008b) Agrochemicals increase trematode infections in a declining amphibian species. Nature 455, 1235–1240. 18972018 10.1038/nature07281

[ref49] Rohr JR, Sack A, Bakhoum S, Barrett CB, Lopez-Carr D, Chamberlin AJ, Civitello DJ, Diatta C, Doruska MJ, De Leo GA, Haggerty CJE, Jones IJ, Jouanard N, Lund AJ, Ly AT, Ndione RA, Remais JV, Riveau G, Schacht AM, Seck M, Senghor S, Sokolow SH and Wolfe C (2023) A planetary health innovation for disease, food and water challenges in Africa. Nature 619, 782–787. 37438520 10.1038/s41586-023-06313-z

[ref50] Rudko SP, Reimink RL, Froelich K, Gordy MA, Blankespoor CL and Hanington PC (2018) Use of qPCR-based cercariometry to assess swimmer's itch in recreational lakes. EcoHealth 15, 827–839. 30120669 10.1007/s10393-018-1362-1 PMC6267424

[ref51] Rudko SP, McPhail BA, Reimink RL, Froelich K, Turnbull A and Hanington PC (2022) Non-resident definitive host presence is sufficient to sustain avian schistosome populations. International Journal for Parasitology 52, 305–215. 35007566 10.1016/j.ijpara.2021.11.010

[ref52] Sckrabulis JP (2020) *Environmental Predictors of Snail-Borne Parasitism* (Ph.D., Oakland University). In ProQuest Dissertations and Theses (2479810303). ProQuest Dissertations & Theses global.

[ref53] Sckrabulis JP, Flory AR and Raffel TR (2020) Direct onshore wind predicts daily swimmer's itch (avian schistosome) incidence at a Michigan beach. Parasitology 147, 431–440. 31965949 10.1017/S0031182020000074 PMC10317682

[ref54] Skírnisson K, Galaktionov KV and Kozminsky EV (2004) Factors influencing the distribution of digenetic trematode infections in a mudsnail (*Hydrobia ventrosa*) population inhabiting salt marsh ponds in Iceland. Journal of Parasitology 90, 50–59. 15040666 10.1645/GE-118R

[ref55] Soldánová M, Selbach C and Sures B (2016) The early worm catches the bird? Productivity and patterns of *Trichobilharzia szidati* cercarial emission from *Lymnaea stagnalis*. PLoS ONE 11, e0149678. 26895541 10.1371/journal.pone.0149678 PMC4760985

[ref56] Soldánová M, Born-Torrijos A, Kristoffersen R, Knudsen R, Amundsen P and Scholz T (2022) Cercariae of a bird schistosome follow a similar emergence pattern under different subarctic conditions: first experimental study. Pathogens (Basel, Switzerland) 11, 647. 35745501 10.3390/pathogens11060647 PMC9227376

[ref57] Soper DM, Raffel TR, Sckrabulis JP, Froelich KL, McPhail BA, Ostrowski MD, Reimink RL, Romano D, Rudko SP and Hanington PC (2023) A novel schistosome species hosted by *Planorbella* (*Helisoma*) *trivolvis* is the most widespread swimmer's itch-causing parasite in Michigan inland lakes. Parasitology 150, 88–97. 10.1017/S0031182022001561 PMC10090624 36349562

[ref58] Tiemann JS Cummings KS and Mayer CA (2011) Distribution of Pleurocerids (Gastropoda) of Illinois. PDFs: Snail Ecology No. 9. Springfield, IL, USA: Illinois Department of Natural Resources.

[ref59] Underwood GJC, Thomas JD and Baker JH (1992) An experimental investigation of interactions in snail-macrophyte-epiphyte systems. Oecologia 91, 587–595. 28313514 10.1007/BF00650335

[ref60] Upatham ES (1974) Dispersion of St. Lucian *Schistosoma mansoni* cercariae in natural standing and running waters determined by cercaria counts and mouse exposure. Annals of Tropical Medicine and Parasitology 68, 343–352. 4447392 10.1080/00034983.1974.11686957

[ref61] Verbrugge LM, Rainey JJ, Reimink RL and Blankespoor HD (2004a) Prospective study of swimmer's itch incidence and severity. Journal of Parasitology 90, 697–704. 15357056 10.1645/GE-237R

[ref62] Verbrugge LM, Rainey JJ, Reimink RL and Blankespoor HD (2004b) Swimmer's itch: incidence and risk factors. American Journal of Public Health 94, 738–741. 15117691 10.2105/ajph.94.5.738 PMC1448328

[ref63] Warren KS and Peters PA (1968) Cercariae of *Schistosoma mansoni* and plants: attempt to penetrate *Phaseolus vulgaris* and *Hedychium coronarium* produces a cercaricide. Nature 217, 647–648. 5637738 10.1038/217647a0

[ref64] Wood CL, Sokolow SH, Jones IJ, Chamberlin AJ, Lafferty KD, Kuris AM, Jocque M, Hopkins S, Adams G, Buck JC, Lund AJ, Garcia-Vedrenne AE, Fiorenza E, Rohr JR, Allan F, Webster B, Rabone M, Webster JP, Bandagny L, Ndione R, Senghor S, Schacht AM, Jouanard N, Riveau G and De Leo GA (2019) Precision mapping of snail habitat provides a powerful indicator of human schistosomiasis transmission. Proceedings of the National Academy of Sciences 116, 23182–23191. 10.1073/pnas.1903698116 PMC6859407 31659025

[ref65] Yang GJ, Gemperli A, Vounatsou P, Tanner M, Zhou XN and Utzinger J (2006) A growing degree-days based time-series analysis for prediction of *Schistosoma japonicum* transmission in Jiangsu province, China. The American Journal of Tropical Medicine and Hygiene 75, 549–555. 16968940

[ref66] Zhou YB, Yang MX, Yihuo WL, Liu GM, Wang HY, Wei JG and Jiang QW (2011) Effect of habitat fragmentation on the schistosome-transmitting snail *Oncomelania hupensis* in a mountainous area of China. Transactions of the Royal Society of Tropical Medicine and Hygiene 105, 189–196. 21367442 10.1016/j.trstmh.2010.12.006

