# Supplementary material for: Large-scale spatial drivers of avian schistosomes in Northern Michigan inland lakes
Source: Parasitology. 2024 Mar 11;151(5):495–505. doi: 10.1017/S0031182024000337 (PMC11106508; doi:10.1017/S0031182024000337)
Supplement: Sckrabulis et al. supplementary material [file S0031182024000337sup001.docx]

Large-scale spatial drivers of avian schistosomes in Northern Michigan inland lakes

Jason P. Sckrabulis^1*^, Madelyn L. Messner^1^, Jenna Stanny^1^, Ryan B. McWhinnie^1^, Hamzah D. Ansari^2^, Aleena M. Hajek^1^, Alexander Bageris^1^, Thomas R. Raffel^1^

^1^ Department of Biological Sciences, Oakland University, 118 Library Drive, 374 Dodge Hall, Rochester, Michigan USA 48309

^2^ Department of Chemistry, Oakland University, 146 Library Drive, 260 Mathematics and Science Center, Rochester, Michigan USA 48309

^*^ Corresponding author: jason.sckrabulis@gmail.com; ORCID: 0000-0002-6901-094X

^*^ Present address: Department of Biological Sciences, University of Notre Dame, 100 Galvin Life Science Center, Notre Dame, Indiana USA 46556

**Supplementary Materials**

*Low-cost protocol for avian schistosome DNA detection*

Preserved samples were centrifuged at 1500 RPM for 2 minutes to concentrate the sample for removal of ethanol preservative. The remaining ethanol evaporated off in a 55°C drying oven, and samples were frozen until DNA extraction. DNA was extracted using a Proteinase K extraction procedure with bead beating following a modified version of Qiagen’s DNA isolation from mouse tail tissue (MA08, Qiagen 2010). To cut costs, we mixed our own lysis buffer (0.1 M Tris pH 8.5, 0.2 M NaCl, 5 mM EDTA, 0.4% SDS) and used 0.5 grams of acid-washed glass sandblasting abrasive for bead beating. Extractions were carried out using 1 mL of lysis buffer and 10 μL of Proteinase K unless samples had more than 1 gram of initial sediment/debris, in which case we added additional extraction reagents to maintain a ratio of 1 mL lysis buffer + 10 μL Proteinase K per 1 g sample. Samples were securely capped and placed in a 55°C water bath and vortexed for 5 seconds every 15 minutes for 2 hours. After the 2-hour extraction process, samples were centrifuged at 1500 RPM for 2 minutes, and supernatant containing potential schistosome DNA was pipetted into a pre-labeled 1.5 mL microcentrifuge tube and stored at -20°C.

We used a real-time qPCR TaqMan assay based on the assay described by Jothikumar et al. (2015), who designed primers to target 18S ribosomal RNA (rDNA) gene sequences based on a diverse panel of schistosome isolates representing 13 genera and 20 species. We utilized the custom primers and probe described by Jothikumar et al. (2015), which were designed to target a highly conserved region to maximize detection of itch-causing cercariae. The TaqMan probe was labeled with FAM (6-Carboxyfluorescein) at the 5’ end and black hole quencher (BHQ-1) at the 3’ end. The TaqMan real-time PCR assay consisted of a 25 μL final reaction volume containing TaqMan Universal PCR Master Mix with 0.25 μM of each forward and reverse primer, 0.1 μM TaqMan probe, and 5 μL DNA sample (1:100 dilution). We added ~1.4 μg/μL of bovine serum albumin (BSA) to our PCR reactions to help reduce inhibition due to humic substances (Garland et al., 2010). All reactions also contained a TaqMan Exogenous Internal Positive Control (IPC) primer-probe and template to allow us to assess PCR reaction inhibition (Hyatt et al., 2007; Garland et al., 2010). All amplification reactions for the TaqMan assay were performed on Bio-Rad CFX Connect Real-time PCR Detection System using the CFX Manager Software 3.1 (Bio-Rad Laboratories Inc., Hercules, CA, USA).

Quantitation cycles (Cq values) were recorded for each unknown sample and were compared to a standard curve to calculate the estimated number of cercariae per liter of water. Standards were created by counting 5 *Trichobilharzia* spp. cercariae using microscopy, and then subjecting them to the DNA extraction procedure outlined above. The standards often exhibited reaction inhibition at higher concentrations, probably due to the presence of increased concentrations of target DNA. It was not possible to reduce reaction inhibition in standards by dilution, because this would have simply generated lower-concentration standards. To generate a more accurate (less biased) standard curve, we corrected for inhibition of standards mathematically, using the difference in IPC cycle threshold (Ct) score for each reaction relative to the negative control wells for each plate. There was no significant among-plate variation in the standards, so we pooled them to generate a single standard curve.

In our preliminary survey in 2015, all filtered water samples were assayed in singlicate to control costs (M. L. Messner, unpublished). For the current survey, we decided to divide each field sample in half prior to extraction, which we hoped would allow us to assess potential sources of error in the assay (i.e., extraction error versus qPCR error). We ran qPCR in singlicate for each extracted DNA sample (i.e., two qPCR reactions per field sample), using a 1:500 dilution. We chose this level of dilution based on our experience from the 2015 survey, in which we frequently observed reaction inhibition at 1:100 dilution and had to re-run those samples at 1:500 (M. L. Messner, unpublished). Reaction inhibition can be caused by humic acids present in the sample, or by excessive amounts of target DNA, and both sources of inhibition can be reduced by diluting the sample, reducing measurement bias. We decided not to dilute further than 1:500 (e.g., 1:1000) because we found that this led to an unacceptable loss of assay precision, presumably due to a decrease in the number of gene copies present in positive samples, and because evidence for reaction inhibition was rare at a 1:500 dilution. It is important to note that the assay would have been more precise using more concentrated samples (i.e., 1:10 or 1:100 dilutions). However, we decided that reducing sources of bias was more important than maintaining high assay precision, partly because we were primarily interested in among-site variation in cercaria abundance. Low assay precision is less of a concern if you are planning to analyze the average of 28 samples (i.e., a minimum of 56 qPCR reactions per sampling site).

We re-ran samples through qPCR if there was a substantial difference between the two “duplicate” reactions, or if there was evidence of inhibition from the IPC reaction or some other problem (e.g., a non-sinusoidal reaction curve). We discounted and re-ran a small number (< 10) of inhibited or obviously spurious reactions, and otherwise calculated the averaged the Ct score for each extracted sample that was assayed multiple types. For each field sample, we added together the qPCR estimates of cercaria abundance for the two extractions. qPCR data is typically lognormally distributed, so we log_10_-transformed the daily cercaria estimates and averaged them to obtain log_10_ cercaria abundance for each site. Log_10_ cercaria abundance was used as our proxy for avian schistosome risk for all our among-site analyses.

*Field survey methods*

*Dreissena spp. (mussel) sampling*

*Dreissena* spp. (zebra and quagga mussel) settling rates were measured by placing two samplers (Fig. S3) at each site in July and leaving them undisturbed through September, to allow sufficient time for new mussels settle and attach onto available substrates (Mackie et al., 1990). We suspended two samplers in the water column at each site, either hanging from an existing dock structure or from a buoy. Mussel samplers were based on a published design (Herman and Wickman, 2014) and comprised of a stacked array of three roughened PVC plastic sheets (Fig. S3). Samplers were collected in October. They were disassembled and scraped free of zebra mussels, which were preserved in 70% ethanol for analysis of wet mass and approximate counts. To determine the approximate number of mussels on each sampler, we massed 10 randomly selected mussels from each sampler. We then divided the total mussel biomass per sampler by the mean mass per mussel to estimate the total number of mussels settled.

*Crayfish trapping*

At each site, we conducted two crayfish trapping sessions spaced two weeks apart. For each trapping session, we set three traps overnight, for a total of six trapping nights at each site. Traps were baited with tuna using cages made from tea diffusers and string, positioning the bait in the middle of the trap. We used two crayfish traps (2-inch diameter opening) and one minnow trap (1-inch diameter opening) on each sampling occasion to obtain data on both large and small crayfish. All crayfish caught were documented with photographs.

*Periphyton growth*

Three plexiglass tiles (10×10 cm) were sanded, rinsed, and anchored above the benthos at a depth of ~30 cm below the surface (Fig. S4). After three weeks, tiles were removed, placed in pans, and carefully brushed clean of periphyton. For two tiles, vacuum filtration was used to concentrate the sample onto a GF/F glass microfiber filter (0.7 μm; Whatman Inc., Kent, UK). Filters were stored in foil envelopes at -20°C until fluorometric analysis. Filters were incubated for 24 hours in the dark in a 90% methanol solution to promote algal cell lysis following a modified version of the EPA method 445.0 (Arar and Collins, 1997). Fluorometric analysis (Synergy H1 microplate reader, Biotek, Winooski, VT, USA) was used to determine chlorophyll-a levels in relative fluorescence units; fluorescence (emission) was recorded at the 680 nm detection wavelength using an excitation wavelength of 440 nm. We calculated the average fluorescence for two tiles to obtain an index of periphyton growth potential at each site. Algae from the third tile were preserved in Lugol’s solution for possible future analysis of taxonomic composition.

*Zooplankton sampling*

We sampled for zooplankton on three occasions at each site. Each sample consisted of three 5-m horizontal drags with a standard 8-inch zooplankton net. Samples were preserved in 50 mL Lugol’s iodine solution for later analysis. We sub-sampled 2 mL from each sample, counted the three most abundant taxa (class Copepoda, Cladocera, and Ostracoda), and took notes of other rare taxa.

*Water and sediment chemistry*

We collected paired water grab samples at two time points from each site, using acid-washed 250 mL brown HDPE sample bottles. These were rinsed three times with lake water and then filled at a water depth of 50 cm during each sampling event. Samples were placed on ice and frozen within 24 hours and stored at -20°C until nutrient analysis could be completed. Samples were assayed for orthophosphate (EPA 365.1), nitrate/nitrite (EPA 353.2), and ammonia (EPA 350.1) using an AQ1 Discrete Analyzer (Seal Analytical Inc., Mequon, WI, USA).

We analyzed two sets of water samples for nutrient measurements. We ran the second set of analyses because there were unusual patterns in the nitrogen measurements suggesting there might have been some sample degradation, which could allow some of the nitrates/nitrites to convert to ammonium (or vice versa). In the end, we decided there had been enough degradation so that the only way to obtain useful nitrogen measurements was to add together the nitrate/nitrite and ammonium values, yielding total inorganic nitrogen as our best index of nitrogen loading at each site. We used standard Abraxis ELISA test kits to measure concentrations of two common herbicides (2,4-D and glyphosate) in each water sample (Eurofins Abraxis, Warminster, PA, USA). We were also interested in measuring concentrations of triazine herbicides, carbamate herbicides, and organophosphate insecticides, but after two months of working through possible mass spectrometry analyses, we decided these were going to be too complex and expensive to complete within our budget and time frame.

We conducted two sediment grabs at each site using a Petite Ponar® sampler (Wildco, Yulee, FL, USA), and collected two sediment mini-cores from each grab. We used a standard acid digest procedure to extract phosphorus from each sediment sample, and these were analyzed on an AQ1 Discrete Analyzer (Seal Analytical Inc., Mequon, WI, USA) to generate total sediment phosphorus measurements (EPA 3050B) at each time point. The phosphorus measurements for the first time point also revealed unusual patterns (likely due to degradation as with the water chemistry samples); therefore, only the measurements for the later time point are included in our analysis.

*Land use variables*

We assessed the major land use types at the lake-watershed level and within a one-mile perimeter around each lake using a GIS-based watershed-mapping tool. The Long-Term Hydrologic Impact Assessment (L-THIA) model (Lim et al., 2001) is an accessible online tool that assesses the water quality impacts of land use change using data from the 2006 National Land Cover Database (Fry et al., 2012). We utilized the L-THIA for the Great Lakes Watershed Management System to generate percent land use data for the watershed of each lake in the survey (12-digit HUC regions), and for lake polygons that encompassed all of the land within a one-mile perimeter of the shoreline. Percent land use was summarized into the following categories: urban, cropland, pastureland, forest, and water; later we combined cropland and pastureland to create an ‘agriculture’ land use category. Urban land use is defined by open space/city park, low-density residential (1/3 to 2 acre lots), high-density residential (townhomes to 1/4 acre lots), commercial/industrial/transportation, and barren land. Agricultural land use includes grassland, pasture/hay, and generalized cropland agriculture. Forested areas are defined as those with deciduous, mixed, or shrub/scrub forest types (Lim et al., 2001).

**References Cited Only in the Online Enhancements**

**Arar, EJ and Collins, GB** (1997) *In vitro determination of chlorophyll a and pheophytin in marine and freshwater algae by fluorescence*. Cincinnati, Ohio, USA: United States Environmental Protection Agency, Office of Research and Development, National Exposure Research Laboratory

**Fry, JA, Xian, G, Jin, S, Dewitz, JA, Homer, CG, Yang, L, Barnes, CA, Herold, ND and Wickham, JD** (2012) Completion of the 2006 National Land Cover database update for the conterminous United States. *Photogrammetric Engineering and Remote Sensing* **77**, 858–864.

**Garland, S, Baker, A, Phillott, AD and Skerratt, LF** (2010) BSA reduces inhibition in a TaqMan assay for the detection of *Batrachochytrium dendrobatidis*. *Diseases of Aquatic Organisms* **92**(2–3), 113–116. DOI: 10.3354/dao02053

**Herman, L and Wickman, S** (2014) Zebra and quagga mussels monitoring protocol. In Lyden T (ed.). *Aquatic Invasive Species Monitoring Manual*. Madison, Wisconsin, USA: Citizen Lake Monitoring Network, University of Wisconsin Extension. pp. 127-153.

**Hyatt, AD, Boyle, DG, Olsen, V, Boyle, DB, Berger, L, Obendorf, D, Dalton, A, Kriger, K, Hero, M, Hines, H, Phillott, R, Campbell, R, Marantelli, G, Gleason, F and Colling, A** (2007) Diagnostic assays and sampling protocols for the detection of *Batrachochytrium dendrobatidis*. *Diseases of Aquatic Organisms* **73**, 175–192. DOI: 10.3354/dao073175.

**Lim, KJ, Engel, BA, Kim, Y, Bhaduri, B and Harbor, J** (2001) Development of the Long-Term Hydrologic Impact Assessment (LTHIA) WWW systems. In Stott DE, Mohtar RH, and Steinhardt GC (eds.). *Sustaining the Global Farm. Selected papers from the 10th International Soil Conservation Organization Meeting held May 24-29, 1999 at Purdue University and the USDA-ARS National Soil Erosion Research Laboratory*. West Lafayette, Indiana, USA: International Soil Conservation Organization. pp. 1018-1023.

**Mackie, GL, Gibbons, WN, Muncaster, BW and Gray, IM** (1990) *The zebra mussel, Dreissena polymorpha: A synthesis of European experiences and a preview for North America*. Ontario, Canada: Ontario Ministry of the Environment, Queen’s Printer for Ontario.

**Qiagen** (2010) Isolation of DNA from mouse tail tissue using the MagAttract(R) DNA tissue M96 kit—MA08. Qiagen, Hilden, Germany.

Table S1: Complete list of lakes and approximate GPS coordinates of each survey sampling site. Full coordinates are available upon correspondence with the author.

| Lake name | Site ID | GPS Coordinates | |
| --- | --- | --- | --- |
|  |  | Latitude | Longitude |
| Crystal | CA | 44.665 | -86.245 |
|  | CC | 44.690 | -86.207 |
|  | OH | 44.646 | -86.093 |
|  | ON | 44.638 | -86.170 |
| Deer | DS | 45.171 | -84.972 |
| Douglas | BS | 45.560 | -84.675 |
|  | BW | 45.588 | -84.726 |
| Elk | BK | 44.886 | -85.362 |
| Glen | DO | 44.855 | -86.013 |
|  | KA | 44.891 | -85.959 |
|  | ME | 44.868 | -85.930 |
| Hamlin | JD | 44.069 | -86.420 |
|  | MB | 44.028 | -86.452 |
|  | PP | 44.051 | -86.456 |
|  | PR | 44.016 | -86.471 |
| Higgins | DH | 44.437 | -84.704 |
|  | GT | 44.496 | -84.699 |
|  | KB | 44.466 | -84.680 |
|  | SS | 44.641 | -84.744 |
| Intermediate | JG | 45.023 | -85.236 |
|  | TP | 45.070 | -85.260 |
| Leelanau | NF | 45.044 | -85.720 |
|  | PS | 45.003 | -85.771 |
| Lime | MA | 44.896 | -85.849 |
| Little Traverse | RC | 44.925 | -85.854 |
| Margrethe | DL | 44.657 | -84.781 |
|  | LB | 44.636 | -84.793 |
|  | SD | 44.627 | -84.786 |
|  | SF | 44.661 | -84.817 |
| Platte | BB | 44.693 | -86.076 |
|  | IN | 44.675 | -86.079 |
|  | RA | 44.695 | -86.120 |
| Portage | NP | 44.366 | -86.238 |
|  | VP | 44.362 | -86.207 |
| Skegemog | KG | 44.809 | -85.346 |
| Walloon | RK | 45.329 | -85.045 |
|  | W2 | 45.265 | -85.002 |
|  | W3 | 45.308 | -84.987 |

Table S2: A summary of the strongest correlations between pairs of variables (correlation coefficient *r* > 0.4 or < -0.4), focusing on relevant response variables.

| Response variable | Predictor | *r* |
| --- | --- | --- |
| Log_10_ cercariae per L | Lake surface area | 0.52 |
|  | Maximum lake depth | 0.53 |
|  | Cobble substrate | 0.44 |
|  | Log_10_ *Lymnaea* | 0.66 |
|  | Log_10_ large *Lymnaea* | 0.64 |
|  | Log_10_ *Lymnaea* + *Physa* | 0.60 |
|  | Log_10_ large *Lymnaea* + large *Physa* | 0.63 |
|  | Log_10_ *Lymnaea* + *Planorbella* | 0.60 |
|  | Log_10_ *Lymnaea* + *Physa* + *Planorbella* | 0.55 |
| Log_10_ *Lymnaea* | Mean night temperature | -0.46 |
|  | Effective fetch | 0.42 |
|  | Lake surface area | 0.50 |
|  | Maximum lake depth | 0.47 |
|  | Deciduous tree abundance | 0.44 |
|  | Total snails | 0.57 |
| Log_10_ *Pleurocera* | Effective fetch | 0.64 |
|  | Log_10_ turbidity | -0.57 |
|  | Cobble substrate | 0.45 |
|  | Log_10_ *Lymnaea* | 0.88 |
| Log_10_ total snails | Effective fetch | 0.61 |
|  | Log_10_ turbidity | -0.61 |
|  | Conifer tree abundance | 0.41 |
|  | Cobble substrate | 0.46 |
| Mean temperature | Effective fetch | -0.50 |
|  | Lake surface area | -0.63 |
|  | Maximum lake depth | -0.69 |
|  | Log_10_ zooplankton | 0.40 |
|  | Mean day temperature | 0.84 |
|  | Mean night temperature | 0.84 |
| Submergent vegetation | Mean night temperature | 0.44 |
|  | Sediment phosphorus | 0.56 |
|  | Chlorophyll-a (periphyton) | 0.48 |

Table S3: Predictor inclusion (“X”) during stepwise model selection for each dependent variable. Predictors are shown in rows, dependent variables in columns.

| Predictor variable | Log_10_ cercariae per L (qPCR) | Log_10_ *Lymnaea* | Log_10_ *Pleurocera* | Log_10_ total snails | Mean temperature | Mean day temperature | Mean night temperature | Submerged vegetation |
| --- | --- | --- | --- | --- | --- | --- | --- | --- |
| Mean day temperature | X | X | X | X |  |  |  | X |
| Mean night temperature | X | X | X | X |  |  |  | X |
| Mean temperature | X | X | X | X |  |  |  | X |
| Slope | X | X | X | X | X | X | X | X |
| Effective fetch | X | X | X | X | X | X | X | X |
| Wave index (volunteer) | X | X | X | X | X | X | X | X |
| Lake surface area | X | X | X | X | X | X | X | X |
| Max. lake depth (ft) | X | X | X | X | X | X | X | X |
| Watershed area | X | X | X | X |  |  |  | X |
| Land to water ratio | X | X | X | X |  |  |  | X |
| % Urbanized (watershed) | X | X | X | X |  |  |  | X |
| % Cropland (watershed) | X | X | X (b) | X |  |  |  | X (d) |
| % Pasture (watershed) | X | X | X | X |  |  |  | X |
| % Forest (watershed) | X | X | X | X |  |  |  | X (d) |
| % Urban (1 mile of lake) | X | X | X (b) | X |  |  |  | X |
| % Cropland (1 mile of lake) | X | X | X | X |  |  |  | X |
| % Pasture (1 mile of lake) | X | X | X | X |  |  |  | X |
| % Forest (1 mile of lake) | X | X | X | X |  |  |  | X |
| 2,4-D Concentration | X | X | X | X |  |  |  | X (c) |
| Log_10_ turbidity | X | X | X | X | X (c) | X | X | X |
| Avg. alkalinity | X | X | X | X |  |  |  | X |
| Orthophosphate | X | X | X | X |  |  |  | X |
| Inorganic nitrogen | X | X | X | X |  |  |  | X |
| Sediment phosphorus | (a) | (a) | (a) | (a) |  |  |  | (a) |
| Woody local riparian (index) | X | X | X | X | X | X | X | X |
| Grassy local riparian | X | X | X | X | X | X | X | X |
| Barren local riparian | X | X | X | X | X | X | X | X |
| Deciduous local riparian | X | X | X | X | X | X | X | X |
| Confider local riparian | X | X | X | X | X | X | X | X |
| Trees local riparian | X | X | X | X | X | X | X | X |
| Buildings (presence/absence) | X | X | X | X | X | X | X | X |
| Park/Beach | X | X | X | X | X | X | X | X |
| Docks | X | X | X | X | X | X | X | X |
| Roads | X | X | X | X |  |  |  | X |
| Lawn | X | X | X | X |  |  |  | X |
| Boulder sediment (index) | X | X | X | X |  |  |  | X |

Table S3—Continued

| Predictor variable | Log_10_ cercariae per L (qPCR) | Log_10_ *Lymnaea* | Log_10_ *Pleurocera* | Log_10_ total snails | Mean temperature | Mean day temperature | Mean night temperature | Submerged vegetation |
| --- | --- | --- | --- | --- | --- | --- | --- | --- |
| Cobble sediment | X | X | X | X |  |  |  | X |
| Gravel sediment | X | X | X | X |  |  |  | X |
| Sand sediment | X | X | X | X |  |  |  | X |
| Silt sediment | X | X | X | X |  |  |  | X |
| Periphyton growth rate | X | X | X | X |  |  |  |  |
| Submerged vegetation (index) | X | X | X | X | X | X (d) | X |  |
| Emergent vegetation | X | X | X | X | X | X | X |  |
| Floating vegetation | X | X | X | X | X | X | X |  |
| Total vegetation | X | X | X | X | X | X | X |  |
| Log_10_ total zooplankton | X | X | X | X |  |  |  |  |
| Log_10_ bird observations | X | X | X | X |  |  |  |  |
| Log_10_ Anseriformes obs. | X | X | X | X |  |  |  |  |
| Log_10_ mussel density (quadrat) | X | X | X | X |  |  |  |  |
| Log_10_ crayfish abundance | X | X | X | X |  |  |  |  |
| Log_10_ total snail density | X |  |  |  |  |  |  |  |
| Log_10_ Lymnaea density | X |  | X |  |  |  |  |  |
| Log_10_ *Physa* density | X | X | X |  |  |  |  |  |
| Log_10_ *Planorbella* density | X | X | X |  |  |  |  |  |
| Log_10_ *Physa* (large) | X |  |  |  |  |  |  |  |
| Log_10_ *Lymnaea* + *Physa* | X |  |  |  |  |  |  |  |
| Log_10_ *Lymn*. + *Physa* (large) | X |  |  |  |  |  |  |  |
| Log_10_ *Pleurocera* | X | X |  |  |  |  |  |  |
| Log_10_ *Lymn*. + *Planorb.* | X |  |  |  |  |  |  |  |
| Log_10_ *Lymn.* + *Physa* + *Planorb.* | X |  |  |  |  |  |  |  |

(a): Incomplete data: left out of stepwise analysis unless a strong single predictor or hypothesized driver; tested in final model during backward selection stage

(b): Removed from final model because effect was driven by influential outlier

(c): Removed from final model because effect direction was more consistent with reverse causality (coefficient sign was opposite the hypothesized direction)

(d): Effect was non-significant in final mixed-effects model

Table S4: Final models for each other response variables discussed in the main text following stepwise model selection. All final models included “Lake” as a random effect. Note that the “Anova” function from the “car” package uses the Kenwood-Roger approximation for estimating degrees of freedom for F-tests, which can result in non-integer values (Fox and Weisberg, 2019).

| Response | Predictor | Coef. | F | Residual df | P(F) |
| --- | --- | --- | --- | --- | --- |
| Log_10_ Total snails | Log_10_ Turbidity^‡^ | -0.60 | 5.2 | 33.99 | 0.029 |
|  | Effective Fetch | 0.12 | 11.5 | 22.45 | 0.003 |
|  | Conifer index | 0.13 | 8.3 | 31.53 | 0.007 |
| Log_10_ *Pleurocera* | Log_10_ Turbidity | -0.99 | 12.2 | 32.87 | 0.001 |
|  | Effective Fetch | 0.11 | 9.2 | 31.15 | 0.005 |
|  | Mean day temperature^*^ | 0.15 | 4.8 | 32.79 | 0.035 |
| Mean night temperature  (minimum) | Lake surface area^†^ | -0.0001 | 9.3 | 11.37 | 0.011 |
| Mean day temperature (maximum) | Lake depth^†^ | -0.01 | 17.4 | 11.81 | 0.001 |
| Mean temperature (overall) | Lake depth^†^ | -0.01 | 28.3 | 11.81 | <0.001 |

^*^Variable with missing data (only missing 1 datapoint for temperatures)

^†^Model has “Lake” as a random effect from a significant predictor of a lake-level variable from model selection

^‡^Predictor became non-significant when Longitude was added to the final model (spatial autocorrelation)

Table S5: Model outputs that changed following addition of Latitude and Longitude as predictor variables. Log_10_ turbidity was removed as a significant predictor of log_10_ total snails, and Buildings was removed as a significant predictor of submerged vegetation, indicating that the apparent effects of these variables could have been due to spatial confoundment. All models included “Lake” as a random effect. Note that the “Anova” function from the “car” package uses the Kenwood-Roger approximation for estimating degrees of freedom for F-tests, which can result in non-integer values (Fox and Weisberg, 2019).

| Response | Predictor | Coef. | F | Residual df | P(F) |
| --- | --- | --- | --- | --- | --- |
| Log_10_ Total snails | Effective fetch | 0.18 | 20.5 | 9.11 | 0.001 |
|  | Conifer index | 0.13 | 9.4 | 33.64 | 0.004 |
|  | Longitude | -0.29 | 13.1 | 6.96 | 0.009 |
| Submerged vegetation | Sediment phosphorus^*^ | 25.16 | 11.8 | 27.84 | 0.002 |
|  | Longitude | -1.15 | 11.0 | 11.60 | 0.006 |

^*^Variable with missing data (missing 1 datapoint for sediment phosphorus)

Table S6: Predictor inclusion in the top ten models with Log_10_ cercaria abundance as a response variable using an “all-subsets” approach (the “exhaustive” method in the “regsubsets” function and sorted by adjusted R^2^; Lumley and Miller, 2020). Here, we cluster biologically related and highly correlated (minimum r = 0.65) predictors together, indicated by alternating shading. Predictors not included in any of the top ten models were omitted from this output.

| Adj. R^2^ | Log_10_ *Lymnaea^*^* | Log_10_ large *Lymnaea* | Total vegetation | Submergent vegetation^*^ | Sediment phosphorus^*^ | Log_10_ crayfish | Cobble | Watershed area | Land:water ratio | Fetch | Perc. crop (watershed) | Perc. crop (within 1 mi) | Perc. urban (watershed) |
| --- | --- | --- | --- | --- | --- | --- | --- | --- | --- | --- | --- | --- | --- |
| 0.742 | X |  |  | X | X | X | X |  |  | X |  |  |  |
| 0.740 | X |  | X |  | X | X | X |  |  | X |  |  |  |
| 0.737 | X |  |  | X | X | X | X | X |  |  |  |  |  |
| 0.733 | X |  | X |  | X | X | X |  | X |  |  |  |  |
| 0.733 | X |  | X |  | X | X | X |  |  |  |  | X |  |
| 0.728 | X |  | X |  | X | X | X | X |  |  |  |  |  |
| 0.728 | X |  | X |  | X | X | X |  |  |  | X |  |  |
| 0.728 | X |  |  | X | X | X | X |  | X |  |  |  |  |
| 0.728 | X |  |  | X | X | X | X |  |  |  |  |  | X |
| 0.727 | X | X | X |  | X | X |  |  |  |  | X |  |  |

^*^Predictor that was significant in the final model from the stepwise model selection procedure (Table 2)

Table S7: Predictor inclusion in the top ten models with Log_10_ *Lymnaea* density as a response variable using an “all-subsets” approach (the “exhaustive” method in the “regsubsets” function and sorted by adjusted R^2^; Lumley and Miller, 2020). Here, we cluster biologically related and highly correlated (minimum r = 0.65) predictors together, indicated by alternating shading. Predictors not included in any of the top ten models were omitted from this output.

| Adj. R^2^ | Deciduous^*^ | Max. lake depth^*^ | Lake surface area | Perc. crop (watershed) | Perc. crop (within 1 mi) | Docks | Total vegetation | Submergent vegetation | Floating vegetation | Woody | Boulder | Log_10_ *Pleurocera* | Log_10_ *Ostracoda* | Perc. pasture (watershed) | Perc. pasture (within 1 mi) | Log_10_ crayfish |
| --- | --- | --- | --- | --- | --- | --- | --- | --- | --- | --- | --- | --- | --- | --- | --- | --- |
| 0.440 | X | X |  | X |  | X | X |  | X |  |  |  |  |  |  |  |
| 0.434 | X |  | X | X |  |  | X |  | X | X |  |  |  |  |  |  |
| 0.430 | X | X |  |  | X | X |  |  |  |  | X |  |  |  |  | X |
| 0.429 | X | X |  |  | X | X |  |  |  | X |  | X |  |  |  |  |
| 0.426 | X | X |  |  | X | X | X |  | X |  |  |  |  |  |  |  |
| 0.425 | X | X |  | X |  | X |  | X | X |  |  |  |  |  |  |  |
| 0.422 | X | X |  |  | X |  |  |  |  |  |  |  | X | X | X |  |
| 0.421 | X | X |  |  | X | X |  | X | X |  |  |  |  |  |  |  |
| 0.420 | X | X |  | X |  | X | X |  |  |  | X |  |  |  |  |  |
| 0.420 | X |  | X | X |  |  | X |  |  | X |  | X |  |  |  |  |

^*^Predictor that was significant in the final model from the stepwise model selection procedure (Table 2)

Table S8: Predictor inclusion in the top ten models with submergent vegetation as a response variable using an “all-subsets” approach (the “exhaustive” method in the “regsubsets” function and sorted by adjusted R^2^; Lumley and Miller, 2020). Here, we cluster biologically related and highly correlated (minimum *r* = 0.65) predictors together, indicated by alternating shading. Predictors not included in any of the top ten models were omitted from this output.

| Adj. R^2^ | Sediment phosphorus^*^ | Perc. crop (watershed) | Perc. crop (within 1 mi) | Cobble | Gravel | Buildings^*^ | Watershed area | Perc. forest (watershed) | 2,4-D | Perc. pasture (within 1mi) | Deciduous | Lawn | Avg. alkalinity | Log_10_ turbidity |
| --- | --- | --- | --- | --- | --- | --- | --- | --- | --- | --- | --- | --- | --- | --- |
| 0.734 | X | X |  | X |  | X | X | X |  |  |  |  |  |  |
| 0.734 | X | X |  | X |  | X | X |  |  |  |  | X |  |  |
| 0.734 | X | X |  |  | X | X | X | X |  |  |  |  |  |  |
| 0.729 | X | X |  | X |  | X | X |  | X |  |  |  |  |  |
| 0.724 | X | X |  | X |  | X | X |  |  | X |  |  |  |  |
| 0.72 | X | X |  |  | X | X |  | X | X |  |  |  |  |  |
| 0.719 | X | X |  | X |  | X | X |  |  |  | X |  |  |  |
| 0.717 | X | X |  | X |  | X | X |  |  |  |  |  |  | X |
| 0.717 | X | X | X | X |  | X | X |  |  |  |  |  |  |  |
| 0.717 | X | X |  |  | X |  |  | X | X |  |  |  | X |  |

^*^Predictor that was significant in the final model from the stepwise model selection procedure (Table 2)

Table S9: Acknowledgement of community scientists who contributed to this study.

| Lake | Name |
| --- | --- |
| Crystal | Al Flory |
|  | Ted Fischer |
|  | Jana Way |
|  | Joel Buzzell |
|  | Shary Grant |
| Deer | Todd Sorenson |
|  | Alec & Dave Sherman |
| Douglas | Curt Blankespoor |
|  | Kira Surber |
| Elk | Bob & Bryce Kingon |
|  | Dean Ginther |
|  | Ruth Bay |
| Glen | John Kassarjian |
|  | Mike Litch |
|  | Denny Becker |
|  | Bill Meserve |
|  | Jack Laitala |
|  | Chris Dorsey Shugart |
| Hamlin | Ginny Hluchan |
|  | Linda & Ted Leibole |
|  | Judi & Ed Cartier |
|  | Paula & Mike Veronie |
|  | Denny Lewis |
|  | Joe Muzzo |
|  | Mara DeChene |
|  | Gail Hanna |
|  | Kathy Grossenbacher |
|  | Jim Gallie |
| Higgins | Jim Vondale |
|  | Charlene Cornell |
|  | Richard Weadock |
|  | John & Susan Osler |
|  | Anne Grein |
|  | Ken Dennings |
|  | Greg Douglas |
|  | Rebekah Gibson |
|  | Sue Gederbloom |
| Intermediate | Steve & Kathy Young |
|  | Jim & Karen Gilleylen |
|  | Scott Zimmerman |
|  | Marcia Collins |
|  | Claude & Joyce Gilkerson |
|  | Sheridan & Bob Haack |
| Leelanau | David Hunter |
|  | John Popa |
|  | John Lutchko |
|  | Nick Fleezanis |
|  | Page Sikes |
| Lime | Dean Manikas |
| Little Traverse | Len Allgaier |
|  | Kristen Race |
| Margrethe | Sandra & Ken Michalik |
|  | Mike Ravesi |
|  | Nancy Atchinson |
| Platte | Wilfred J. Swiecki |
|  | Bob Blank |
|  | Tom & Christian Inman |
|  | Jackie & John Randall |
| Portage | Al Taylor |
|  | Mary Reed |
|  | Tammy Messner |
|  | Ted Lawrence |
| Skegemog | Dave Hauser |
|  | Kathi Gober |
| Walloon | Christine Wedge |
|  | Russ & Kathy Kittleson |
|  | John Markewitz |
|  | Megan Muller-Girard |


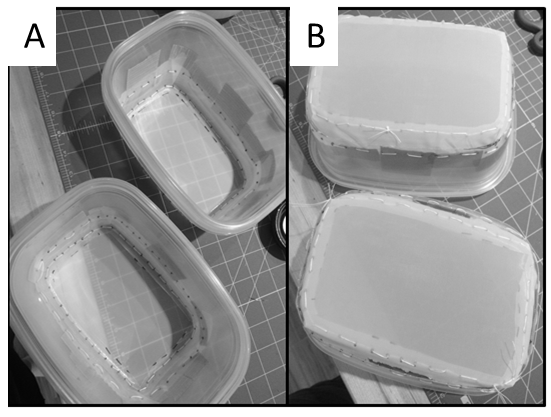


Figure S1: Photographs of the custom-built Nitex filters used for daily cercaria sampling by community science volunteers.





Figure S2: Raw data plots for the main predictors of cercaria abundance (Panels A, C, E), *Lymnaea* density (Panels B & D), and submerged vegetation (Panel F). The top predictors of cercaria abundance were (A) *Lymnaea* spp. density, (C) submerged vegetation, and (E) sediment phosphorus. The top predictors of *Lymnaea* density were (B) maximum lake depth and (D) deciduous tree cover. The top predictors for submerged vegetation were (F) sediment phosphorus and presence of buildings (not shown). All models depict each relationship as a simple linear model of the response and predictor variables.


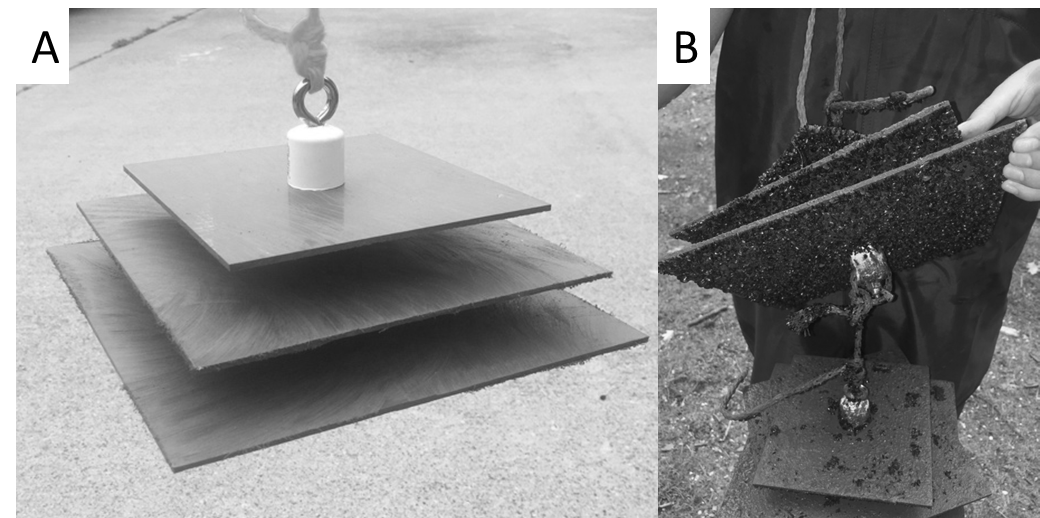


Figure S3: Photographs of PVC mussel settling rate apparatus (A) before deployment, and (B) an example of sample collection after approx. 2 months. The design of the apparatus is based on work by Herman and Wickman (2014).


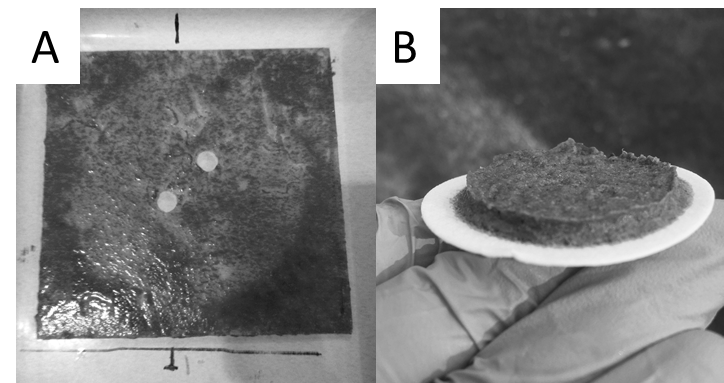


Figure S4: Photographs of the periphyton (attached biofilm) collection method. Panel A depicts a roughened plexiglass tile upon collection. Panel B depicts the results of a particularly heavy biofilm yield after vacuum filtration after removal from the tile.
